# Expression and prognosis analyses of the *Tob/BTG* antiproliferative (*APRO*) protein family in *human* cancers

**DOI:** 10.1371/journal.pone.0184902

**Published:** 2017-09-18

**Authors:** Yuru Bai, Lu Qiao, Ning Xie, Yongquan Shi, Na Liu, Jinhai Wang

**Affiliations:** 1 Department of Gastroenterology, the Second Affiliated Hospital of Xi'an Jiaotong University, Xi'an, Shaanxi Province, China; 2 Xijing Hospital of Digestive Diseases, Xijing Hospital, the Fourth Military Medical University, Xi’an, Shaanxi Province, China; 3 State Key Laboratory of Cancer Biology, the Fourth Military Medical University, Xi’an, Shaanxi Province, China; Centre de Recherche en Cancerologie de Lyon, FRANCE

## Abstract

**Background:**

Despite advances in early diagnosis and treatment, cancer remains the major cause of mortality in the world. The *Tob/BTG* antiproliferative (*APRO*) protein family is reported to participate in diverse human diseases. However, there’s little known about their expression and prognostic values in most human cancers.

**Methods:**

We performed a detailed cancer vs. normal analysis. The mRNA expression levels of *APRO* family in various cancers were analyzed via the Oncomine database. Moreover, the Kaplan-Meier Plotter and PrognScan databases were used to evaluate the prognostic values.

**Results:**

We observed that the mRNA expression levels of *TOB1-2* and *BTG2* were decreased in most cancers compared with normal tissues, while *BTG3* was upregulated in most cancers. In survival analyses based on Kaplan-Meier Plotter, *TOB1*, *BTG1* and *BTG4* showed significant associations with survival outcome of different subtypes of breast cancer. Decreased *BTG2* was related with poor relapse free survival (RFS) in all subtypes of breast cancer. Especially, besides RFS, reduced *BTG2* also indicated worse overall survival and distant metastasis free survival in breast cancer patients who were classified as luminal A. Significant prognostic effects of the whole *APRO* family were also found in lung adenocarcinoma, but not in squamous cell lung carcinoma. In addition, potential correlations between some *APRO* family members and survival outcomes were also observed in ovarian, colorectal and brain cancer.

**Conclusions:**

Some members of *APRO* family showed significant expression differences between cancer and normal tissues, and could be prognostic biomarkers for defined cancer types.

## Introduction

Cancer, a major public health problem, is the leading cause of death worldwide. It’s predicted that there will be 1,688,780 new cancer cases and 600,920 cancer deaths in the United States in 2017 [1]. Although diagnostic techniques and therapeutic methods have been improved, cancer still affects the quality of patients’ life seriously, resulting in serious social and economic burden. It is thus urgently needed to explore the underlying mechanisms of cancer, as well as to identify potential biomarkers to improve diagnosis, therapy and prognosis.

In the early 1990s, several genes which share a high degree of N-terminal sequences homology were found to play important roles in regulating cell proliferation. This family is now referred to as the *Tob/BTG* antiproliferative (*APRO*) protein family. It contains six members in human, namely *TOB1/TOB*, *TOB2*, *BTG1*, *BTG2/PC3/TIS21*, *BTG3/ANA* and *BTG4/PC3B* [2]. Over the past decades, researches have provided evidence that proteins of this family are involved in regulating tissue growth and development by negatively regulating cell cycle. They also interact with transcription factors as transcriptional repressors or enhancers to alter the outcome of DNA binding [3, 4]. Another important role of these proteins is to participate in mRNA decay. The interactions with *CAF1* [5], the *CNOT7* subunit of the *Ccr4-Not* complex [6], or the poly (A)-binding protein *PABPC1* [7], are reported to contribute to this function. Furthermore, it should be noted that *APRO* family participates in regulating proliferation, apoptosis, invasion and metastasis of various cancers [8–10]. Studies also reveal that some members of this family may have significant prognostic effects on human cancers [11, 12].

Taken together, these findings suggest that members of *APRO* family may act as potential therapeutic targets or prognostic biomarkers in some cancers. However, a systematic study about the transcriptional expression and prognostic values in human cancers is still lacking. In the current study, we explored the mRNA expression differences of *APRO* family members between cancer and normal tissues in human cancers via the Oncomine database. Additionally, we assessed the prognostic values using the Kaplan-Meier Plotter and PrognScan databases.

## Materials and methods

### Oncomine database analysis

We used Oncomine (http://www.oncomine.org), an online microarray database, to analyze the mRNA expression differences of *APRO* family between tumor and normal tissues in common human cancers. For each cancer and gene, the thresholds were set as follows: *p*-value: 0.01; fold change: 2; gene rank: 10%; analysis type: cancer vs. normal analysis; data type: mRNA. Cancers, genes, datasets, sample sizes, fold change, *t*-test and *p*-value were obtained from studies that showed statistically differences.

### Kaplan-Meier Plotter database analysis

The KM Plotter (http://kmplot.com/analysis/), which is capable to assess the effect of 54,675 genes on survival using 10,461 cancer samples, including 5,143 breast and 2,437 lung cancers, was applied to evaluate the prognostic values of *APRO* family in these three cancers. For each gene, cancer patients were split into high and low expression group by the median values of mRNA expression. Then the desired probe ID was separately entered into the database. After that, survival analyses were carried out to achieve Kaplan-Meier plots. *P*-value < 0.01 was considered to indicate a statistically significant result. Cancer types, genes, affymetrix ID, survival outcome, HRs, 95% CIs and *p*-values were summarized from the KM plotter webpage; some representative plots were also displayed.

### PrognScan database analysis

PrognScan (http://www.abren.net/PrognoScan/), a new database for meta-analysis of the prognostic value of genes, was utilized to assess the prognostic effects of *APRO* family in other types of cancers. *P*-value < 0.01 was considered to indicate a statistically significant result. The results were downloaded from PrognScan database, cancer types, genes, dataset, probe ID, HRs, 95% CIs and *p*-values were extracted in tables.

## Results

### The mRNA expression levels of *APRO* family in human cancers

**Fig 1:**
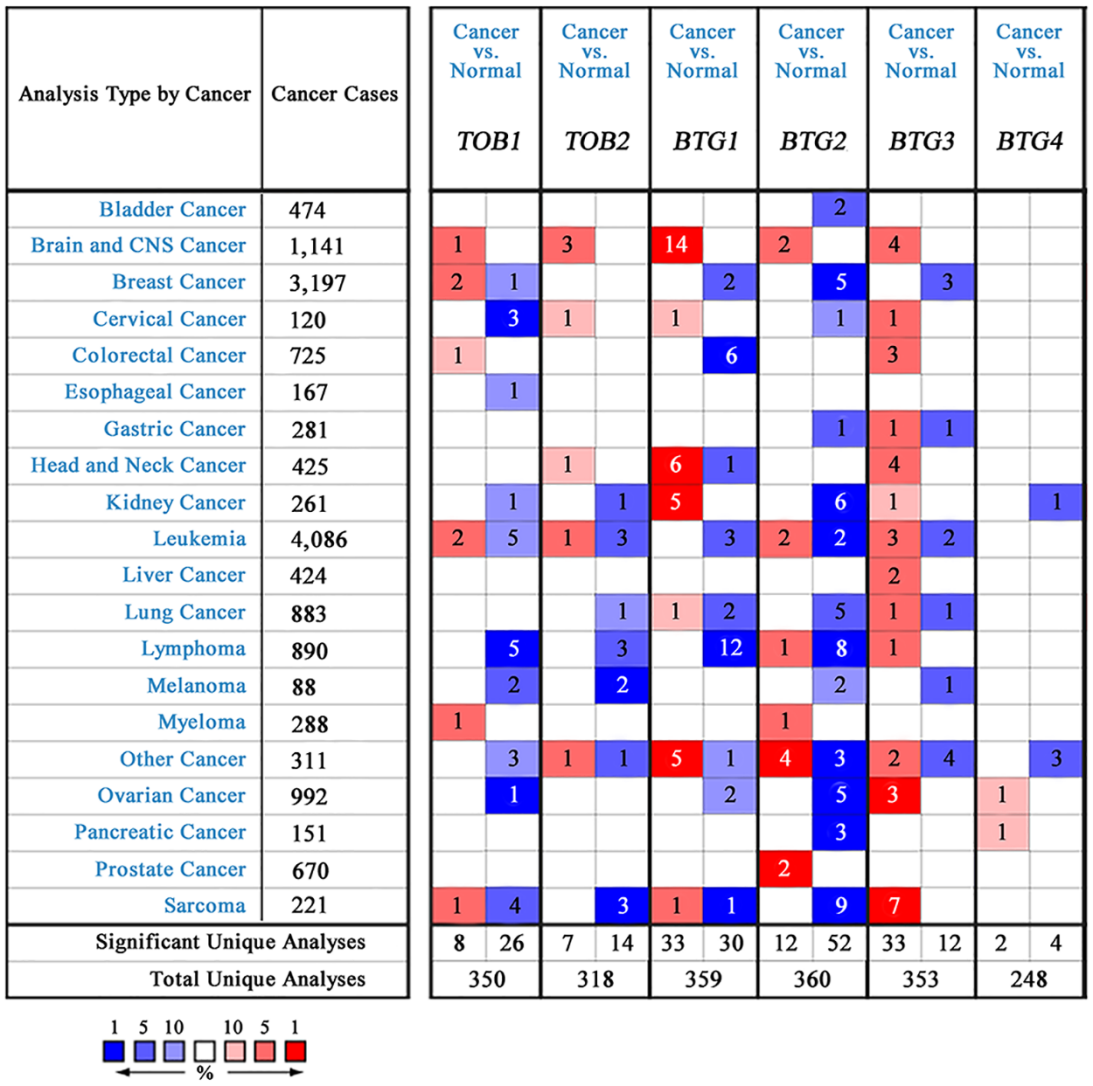
The mRNA expression levels of *APRO* family in human cancers. The number in the colored cell represents the number of analyses meeting thresholds. Cell color is determined by the gene rank. The more intense red (over-expression) or blue (under-expression) indicates a more highly significant over-expressed or under-expressed gene.

To address the mRNA expression differences of *APRO* family between tumor and normal tissues in multiple cancers, we performed an analysis using the Oncomine database. As shown in Fig 1, the database contained a total of 350, 318, 359, 360, 353 and 248 unique analyses for *TOB1*, *TOB2*, *BTG1*, *BTG2*, *BTG3* and *BTG4*, respectively. In 34 studies, *TOB1* was ranked within the top 10% of all genes showing significant statistical differences, 26 of which revealed lower expression levels in tumor than normal tissues, while eight analyses indicated an opposite result. Downregulation of *TOB2* was found in cancers based on 14 studies but overexpressed in seven analyses. Sixty-three significant unique analyses revealed that the mRNA expression level of *BTG1* varied with the type of tumor. Compared to normal tissues, *BTG2* was reduced in tumors, demonstrated by 52 analyses involving 13 kinds of cancers, only 12 studies showed an increased level. Higher expression of *BTG3* was found in most cancers. As for *BTG4*, only six datasets were listed, and the result was opposite. Altogether, the transcriptional expression levels of *TOB1-2* and *BTG2* were significantly reduced in most cancers compared with normal tissues, while *BTG3* was upregulated in most cancers.

The latest data from the American Cancer Society (ACS) show that there are totally 1,688,780 cases of cancers expected to occur in the United States in 2017. For men, the three most commonly diagnosed cancers are prostate, lung and bronchus, and colorectum, which are expected to account for 42% of all new cancer diagnoses in men. Breast, lung and bronchus, and colorectal cancers are the top three cancers to be diagnosed in women, which collectively represent one-half of all cases. Therefore, we focus on the expression and prognosis of *APRO* family in these four tumors, as well as some other common solid tumors [1].

### The expression level and prognostic value in breast cancer

We utilized Oncomine to explore the expression of *APRO* family in ductal and invasive breast carcinoma. A total of 12 datasets were involved in the analysis. With regard to *TOB1*, three of 12 analyses revealed significant difference between cancer and normal groups. In Ma’s dataset [13], we found that *TOB1* was more highly expressed in both ductal breast carcinoma in situ and invasive ductal breast carcinoma, while we obtained an opposite conclusion from Finak’s dataset [14]. According to TCGA database, *BTG1* was found downregulated in invasive ductal breast cancer. Several databases including TCGA and Sorlie [15, 16], indicated a lower expression level of *BTG2* in ductal and invasive breast cancer. *BTG3* was decreased in invasive ductal and lobular breast carcinoma in studies from Finak [14] and Turashvili [17]. For *TOB2* and *BTG4*, there were no significant differences between cancer and normal tissues. All of the statistically significant results were summarized in Table 1.

**Table 1:** Datasets of *APRO* family in breast cancer.

*Gene*	*Dataset*	*Normal (Cases)*	*Tumor (Cases)*	*Fold change*	*t-Test*	*p-value*
***TOB1***	Finak	Breast (6)	Invasive Breast Carcinoma (53)	-3.833	-16.002	3.17E-16
	Ma 4	Breast (14)	Ductal Breast Carcinoma in Situ (9)	2.131	4.337	1.49E-04
		Breast (14)	Invasive Ductal Breast Carcinoma (9)	2.033	4.02	3.19E-04
***BTG1***	TCGA	Breast (61)	Invasive Ductal Breast Carcinoma (389)	-2.102	-15.416	1.95E-30
***BTG2***	TCGA	Breast (61)	Invasive Ductal Breast Carcinoma (389)	-2.586	-11.531	2.51E-20
	Sorlie	Breast (4)	Ductal Breast Carcinoma (64)	-3.244	-5.14	2.00E-03
	Sorlie 2	Breast (4)	Ductal Breast Carcinoma (89)	-3.106	-5.6	2.00E-03
***BTG3***	Finak	Breast (6)	Invasive Breast Carcinoma (53)	-3.354	-11.116	4.12E-16
	Turashvili	Ductal Breast Cell (10)	Invasive Ductal Breast Carcinoma (5)	-4.396	-3.521	3.00E-03
		Lobular Breast Cell (10)				
		Ductal Breast Cell (10)	Invasive Lobular Breast Carcinoma (5)	-4.487	-3.451	4.00E-03
		Lobular Breast Cell (10)				

**Fig 2:**
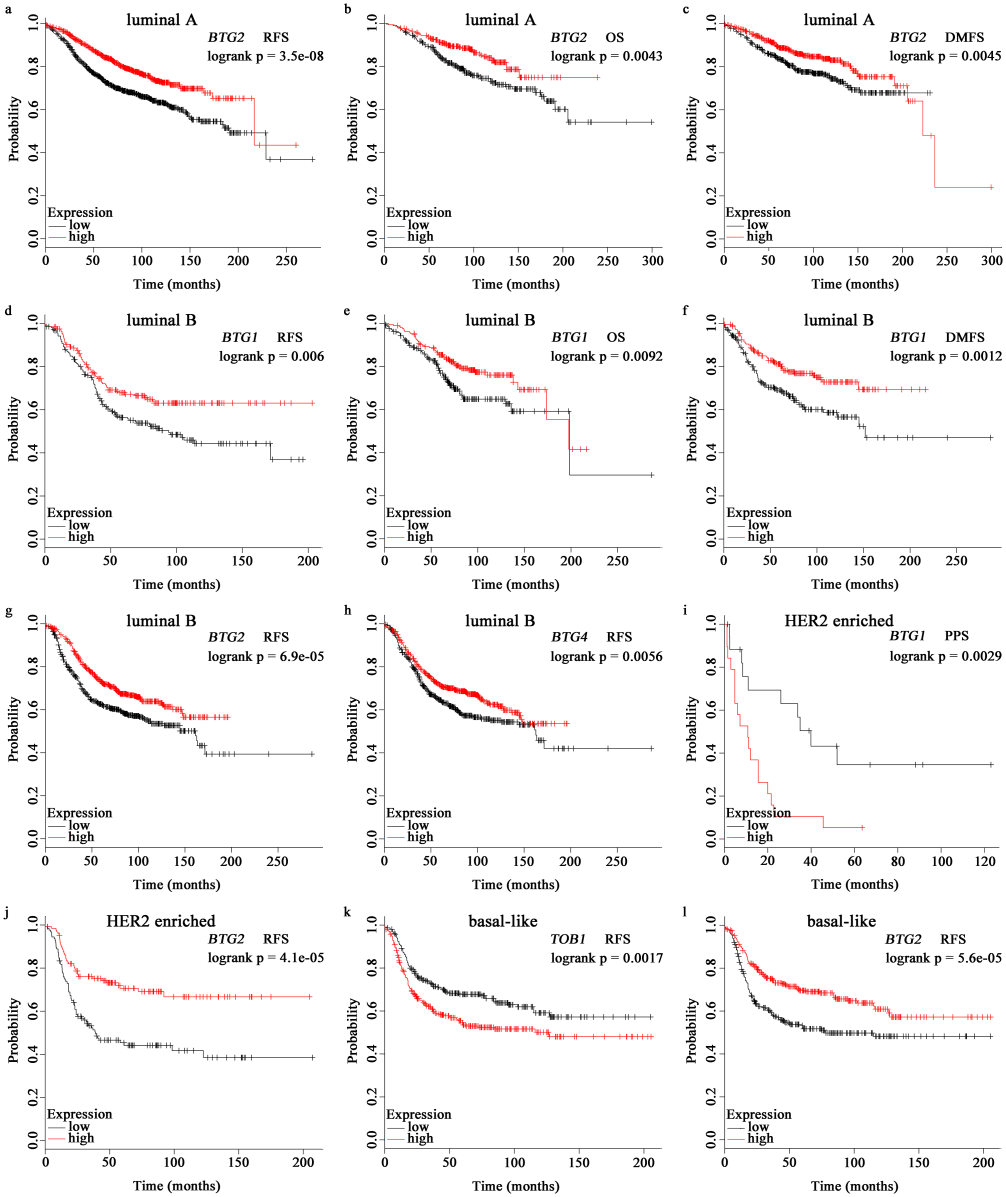
Survival analyses of *APRO* family in breast cancer. OS, overall survival; RFS, relapse free survival; DMFS, distant metastasis free survival; PPS, post progression survival. (a-c): prognosis analysis of *BTG2* in luminal A patients. (d-h): prognosis analysis of *BTG1* in luminal B patients. (i-j): prognosis analyses of *BTG1-2* in HER2+ patients. (k-l): prognosis analyses of *TOB1* and *BTG2* in basal-like patients.

The breast oncology community now describes breast cancer in terms of intrinsic biologic subtypes, and at least four subtypes are defined: basal-like (ER-/PR-/HER2-), luminal A (ER+/HER2-/grade 1 or 2), luminal B (ER+/HER2-/grade 3) and HER2 enriched (any HER2+ tumor). As a result, critical treatment decisions hinge on these molecular findings [18]. Therefore, we investigated prognosis analysis based on these four intrinsic subtypes using the KM Plotter [19]. In particular, decreased *BTG2* (Fig 2a, 2g, 2j and 2l) was associated with poor relapse free survival (RFS) in all subtypes patients. Besides, reduced *BTG2* (Fig 2b and 2c) indicated worse overall survival (OS) and distant metastasis free survival (DMFS) in patients who were classified as luminal A. Higher *BTG1* (Fig 2d–2f) showed better RFS, OS and DMFS in patients with luminal B, while worse post progression survival (PPS) in HER2+ patients (Fig 2i). High expression of *TOB1* (Fig 2k) and low expression of *BTG4* (Fig 2h) was related to worse RFS in basal-like and luminal B patients, respectively. All of the results are summarized in Table A in S1 File.

### The expression level and prognostic value in lung cancer

Equally, using the Oncomine database we analyzed the transcriptional expression of *APRO* family in lung adenocarcinoma and squamous cell lung carcinoma, which account for the majority of lung cancer. According to Selamat [20] and Okayama’s analyses [21], *BTG1* was lower in lung adenocarcinoma, but higher in squamous cell lung carcinoma in Talbot’s study [22]. In a group of datasets including Beer [23], Selamat [20], Su [24], Hou [25] and Wachi [26], *BTG2* was downregulated in both lung adenocarcinoma and squamous cell lung carcinoma. In Talbot’s dataset [22], it was extracted that *BTG3* was elevated in squamous cell lung carcinoma compared to normal group. None of the datasets revealed statistically differences between lung cancer and normal tissue groups for *TOB1* and *BTG4*. The details were shown in Table 2.

**Table 2:** Datasets of *APRO* family in lung cancer.

*Gene*	*Dataset*	*Normal (Cases)*	*Tumor (Cases)*	*Fold change*	*t-Test*	*p-value*
***BTG1***	Selamat	Lung (58)	Lung Adenocarcinoma (58)	-2.037	-9.67	1.04E-15
	Okayama	Lung (20)	Lung Adenocarcinoma (226)	-2.011	-8.078	5.38E-09
	Talbot	Lung (2)	Squamous Cell Lung Carcinoma (34)	2.101	5.75	1.95E-07
***BTG2***	Beer	Lung (10)	Lung Adenocarcinoma (86)	-7.037	-6.881	3.32E-10
	Selamat	Lung (58)	Lung Adenocarcinoma (58)	-3.537	-11.678	2.89E-21
	Su	Lung (30)	Lung Adenocarcinoma (27)	-2.368	-7.144	1.20E-09
	Hou	Lung (65)	Squamous Cell Lung Carcinoma (27)	-2.182	-8.016	1.16E-10
	Wachi	Lung (5)	Squamous Cell Lung Carcinoma (5)	-2.177	-3.663	3.00E-03
***BTG3***	Talbot	Lung (2)	Squamous Cell Lung Carcinoma (34)	2.074	6.541	1.09E-08

**Fig 3:**
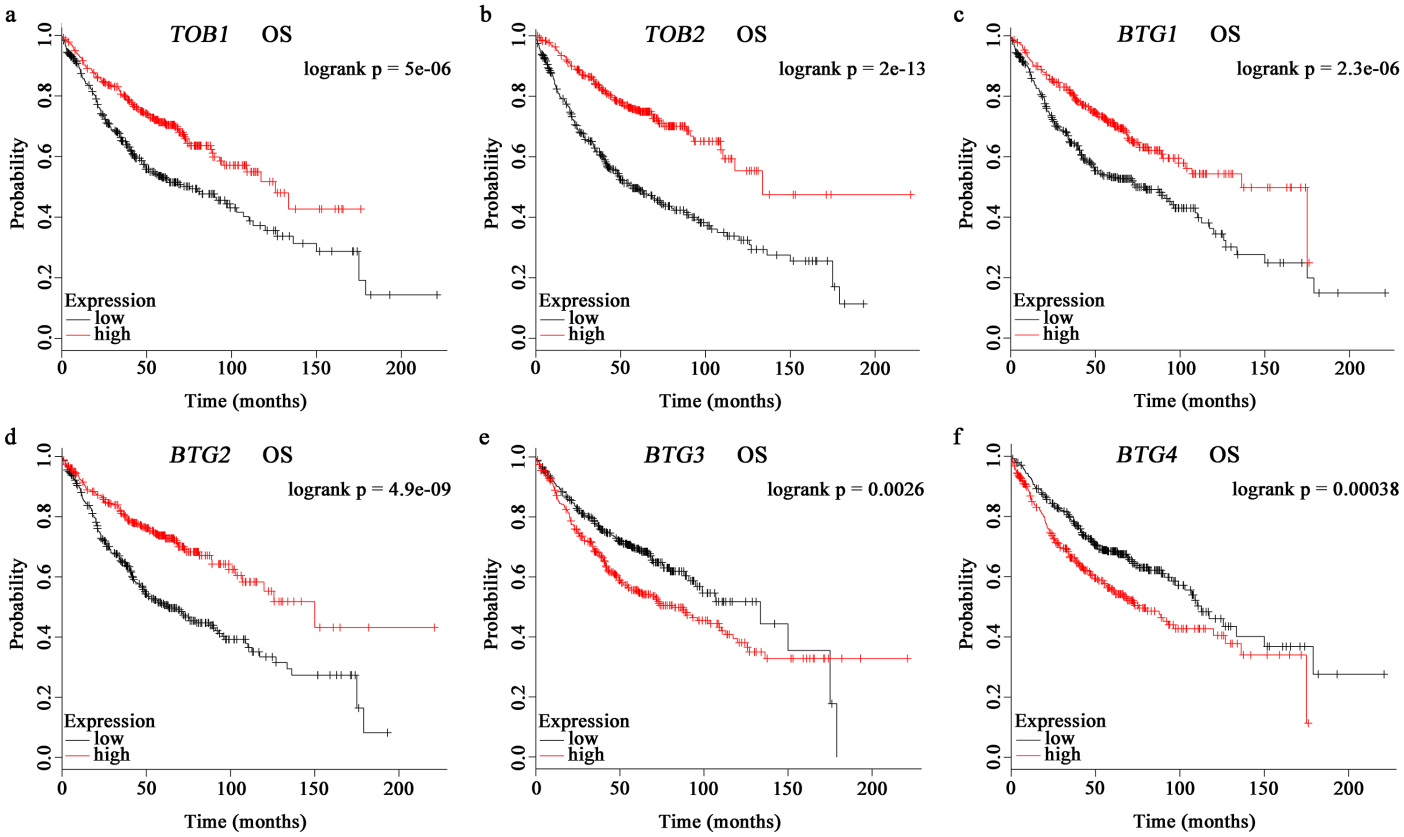
Survival analyses of *APRO* family in lung adenocarcinoma. OS, overall survival. Survival analyses of *TOB1* (a), *TOB2* (b), *BTG1* (c), *BTG2* (d), *BTG3* (e) and *BTG4* (f) were obtained from the Kaplan-Meier Plotter database.

Next, we proceeded to determine whether *APRO* family is associated with the prognosis of lung cancer patients via KM Plotter [27]. Overall survival (OS) and post progression survival (PPS) were studied for each gene. For patients with lung adenocarcinoma, no gene was associated with PPS, while all members of *APRO* family were related to OS. Higher *TOB1-2* (Fig 3a and 3b) and *BTG1-2* (Fig 3c and 3d) implied better OS. On the contrary, increased *BTG3* (Fig 3e) and *BTG4* (Fig 3f) predicted worse OS. Unfortunately, no gene showed statistical significance for squamous cell lung carcinoma patients. All the detailed prognostic analyses are shown in Table B in S1 File.

### The expression level and prognostic value in prostate cancer

There’re totally 16 datasets for prostate cancer in the Oncomine database. Only two datasets showed significant differences between prostate cancer and normal tissues. In Yu’s study [28], we found that *BTG2* was increased in prostate cancer (fold change = 2.258, *t* = 7.407, *p* = 7.27E-11). We came to a consistent conclusion (fold change = 2.575, *t* = 4.238, *p* = 1.03E-04) from the dataset of Wallace [29]. As for other members of *APRO* family, we didn’t observe any expression differences between cancer and normal group.

Afterwards we explored the associations between the *APRO* family and the survival outcome of prostate cancer patients in the PrognScan database [30]. This database provides overall survival (OS) for prostate cancer. However, as shown in Table C in S1 File, there were no statistically significant data.

### The expression and prognostic value in colorectal cancer

As for colon and rectal carcinoma, all statistically significant datasets were extracted in Table 3. It was shown that *TOB1* was increased in rectal mucinous adenocarcinoma from Kaiser’s study [31]. Five comparisons from Skrzypczak’s [32] and TCGA datasets revealed that the expression of *BTG1* was reduced in both colon and rectal cancer compared with normal tissues. According to Skrzypczak’s [32] and TCGA datasets, *BTG3* was elevated in colon carcinoma and rectal mucinous adenocarcinoma. *TOB2*, *BTG2* and *BTG4* had no expression difference between colorectal cancer and normal tissues.

**Table 3:** Datasets of *APRO* family in colorectal cancer.

*Gene*	*Dataset*	*Normal (Cases)*	*Tumor (Cases)*	*Fold change*	*t-Test*	*p-value*
***TOB1***	Kaiser	Colon (5)	Rectal Mucinous Adenocarcinoma (4)	3.354	6.708	8.43E-04
***BTG1***	Skrzypczak 2	Colon (10)	Colon Carcinoma (5)	-2.622	-21.877	2.31E-07
		Colon (10)	Colon Carcinoma (5)	-2.545	-21.7	3.72E-10
	TCGA	Colon (19)/Rectum (3)	Colon Adenocarcinoma (101)	-2.161	-10.903	2.46E-13
		Colon (19)/Rectum (3)	Rectal Adenocarcinoma (60)	-2.398	-11.543	2.56E-15
		Colon (19)/Rectum (3)	Rectal Mucinous Adenocarcinoma (6)	-2.337	-11.166	1.02E-11
***BTG3***	TCGA	Colon (19)/Rectum (3)	Rectal Mucinous Adenocarcinoma (6)	2.225	6.283	4.21E-05
	Skrzypczak 2	Colon (10)	Colon Carcinoma (5)	3.212	9.901	1.28E-05
		Colon (10)	Colon Carcinoma (5)	2.814	9.59	1.53E-07

Numerous survival analyses were included in the PrognScan database [30]. Only lower expression of *BTG4* revealed poor prognosis in colorectal cancer patients (S1 Fig). All of the data are collected in Table D in S1 File.

### The expression levels and prognostic values in other cancers

Subsequently, we elucidated the mRNA expression levels in some other solid tumors showing the highest numbers of significant differences in Fig 1, including kidney, ovarian, brain and CNS cancer. All of the significant analyses of these cancers were shown in Table E in S1 File. As for clear cell renal cell carcinoma, which account for the majority of kidney cancer, *BTG2* and *BTG4* were reduced, while *BTG1* was elevated in clear cell renal cell carcinoma compared with normal kidney tissue group. In ovarian serous adenocarcinoma, the most common type of ovarian cancer, *BTG3*-*4* were upregulated, but *BTG1-2* were found to be at lower expression levels. Intriguingly, *TOB1-2* and *BTG1-3* were at a higher expression level in various types of brain and CNS cancer.

At last, we examined the prognostic significance of *APRO* family in all tumors mentioned above in the PrognScan database [30]. In summary, we did not investigate significant prognostic effects of *APRO* family in kidney cancer. Potential correlations between *APRO* family and survival outcomes were observed in ovarian and brain cancer. Specifically, low expression of *TOB2* was associated with poor overall survival (OS) in ovarian cancer patients (S2a Fig). With respect to brain cancer, increased *TOB1-2* and *BTG3* revealed poor prognosis (S2b–S2d Fig), while higher *BTG2* was related to better prognosis (S2e Fig). Survival analyses of patients with all these solid tumors are shown in Table F in S1 File.

## Discussion

In this study, we systematically analyzed the mRNA expression level of *APRO* family in various types of cancers using the Oncomine database. We focused on the most common types of cancers, including breast, lung, prostate and colorectal cancer, and their most frequent subtypes. In addition, we explored the prognostic values of this family in cancer patients via the Kaplan-Meier Plotter and PrognScan databases.

In clinical samples of patients with breast and thyroid cancer, decreased *TOB1* is frequently detected [33, 34], as well as *BTG2* is reduced in breast and kidney cancer [35, 36]. Consistent with previous studies, our expression analyses showed that the mRNA levels of *TOB1* and *BTG2* were reduced in most cancers compared to normal tissues. As for *TOB2*, there are few current studies on its relationship with cancer. In our study, we observed *TOB2* was at a lower expression level in most cancers. Beside, we found that the mRNA expression level of *BTG1* varied with tumor type, such as lower in breast, colorectal and ovarian cancer and lung adenocarcinoma, higher in kidney, cervical and squamous cell lung cancer. For *BTG3*, except decreased in breast cancer tissues, consistent with previous report [37], the vast majority of cancers showed an increased mRNA expression in our study. In contrast, *BTG3* is reported at a low expression level in some cancer tissues or cells, such as lung cancer [38] and hepatocellular carcinoma [39]. The contradictory results may be due to different sample sources, histological types or detection methods. There were few datasets for *BTG4* in our analyses, but it was proposed interestingly by others that inactivation of *BTG4* may be a contributory factor for colon cancer [40].

In recent years, comprehensive transcriptional profiling studies have revealed four intrinsic biological subtypes of breast cancer, defined as luminal A, luminal B, HER2 enriched and basal-like, which have been shown to be robust for predicting treatment sensitivity and survival outcomes [18]. Interestingly, we discovered that decreased *BTG2* was related to worse relapse free survival (RFS) in all subtypes patients. Especially, besides RFS, reduced *BTG2* also indicated poor overall survival (OS) and distant metastasis free survival (DMFS) in breast cancer patients who were classified as luminal A. Therefore, we propose *BTG2* could act as a prognostic biomarker for breast cancer, especially for the subtype of luminal A.

Lung cancer is the most common cancer and the leading cause of cancer death in China [41] and worldwide [1]. Lung adenocarcinoma and squamous cell lung carcinoma are the most prevalent subtypes of lung cancer. Our prognosis analyses showed that no gene was associated with overall survival (OS) and post progression survival (PPS) for patients with squamous cell lung carcinoma, as well as PPS for patients with lung adenocarcinoma. However, all members of *APRO* family were related to OS in lung adenocarcinoma. Thus, we inferred this family could serve as prognostic biomarkers for lung adenocarcinoma rather than squamous cell lung carcinoma.

In this study, we also found that *TOB1*, *BTG1 and BTG3* could be a prognostic marker or potential therapeutic target in several cancer types, consistent with previous reports [11, 12, 42]. Moreover, we had some other new discoveries that downregulated *TOB1* mRNA expression implied better overall survival (OS) in brain cancer patients. Potential correlation between *TOB2* and survival outcome was also observed in ovarian and brain cancer. *BTG4* was related to survival outcomes in breast and colorectal cancer. Regretfully, it seemed that this family had nothing to do with the survival outcomes of prostate cancer.

Our analyses would contribute to comprehensively understand the expression levels and the prognostic values of the *APRO* family in some solid tumors, as well as provide the evidence that members of this family could be employed as novel prognostic biomarkers or promising therapeutic targets for human carcinomas. Nevertheless, we concentrated on only the mRNA expression levels and the prognostic values of this family, neither their protein expression levels nor some possible signaling pathways were further analyzed. Sample cohort studies are needed to be performed to validate the prognostic values of this family, and many more research should be carried out to explore the underlying molecular mechanisms in tumors.

In summary, we comprehensively analyzed the mRNA expression levels and prognostic values of *APRO* family in most common cancers. Several members including *TOB1-2* and *BTG2-3*, exhibited significant expression differences between cancer and normal tissue groups in defined cancers. Furthermore, we put forward *BTG2* could act as a prognostic biomarker for breast cancer, especially for the subtype of luminal A, as well as this family may be prognostic biomarkers for lung adenocarcinoma.

## Supporting information

S1 File Table A. Survival analyses of *APRO* family in breast cancer. Table B. Survival analyses of *APRO* family in lung cancer. Table C. Survival analyses of *APRO* family in prostate cancer. Table D. Survival analyses of *APRO* family in colorectal cancer. Table E. Datasets of *APRO* family in kidney, ovarian, brain and CNS cancer. Table F. Survival analyses of *APRO* family in kidney, ovarian and brain cancer.
(DOCX)

S1 Fig Survival analyses of *APRO* family in colorectal cancer. DFS, disease free survival. Survival analysis of *BTG4* was obtained from the PrognScan database.
(TIF)

S2 Fig Survival analyses of *APRO* family in ovarian and brain cancer. OS, overall survival. (a): survival analysis of *TOB2* in ovarian cancer. (b-e): survival analyses of *TOB1-2* and *BTG2-3* in brain cancer.
(TIF)
