# Supplementary material for: Expression and prognosis analyses of the Tob/BTG antiproliferative (APRO) protein family in human cancers
Source: PLoS One. 2017 Sep 18;12(9):e0184902. doi: 10.1371/journal.pone.0184902 (PMC5602628; doi:10.1371/journal.pone.0184902)
Supplement: S1 File — Table A. Survival analyses of APRO family in breast cancer. Table B. Survival analyses of APRO family in lung cancer. Table C. Survival analyses of APRO family in prostate cancer. Table D. Survival analyses of APRO family in colorectal cancer. Table E. Datasets of APRO family in kidney, ovarian, brain and CNS cancer. Table F. Survival analyses of APRO family in kidney, ovarian and brain cancer. (DOCX) [file pone.0184902.s001.docx]

**Table A. Survival analyses of *APRO* family in breast cancer.**

| ***Gene*** | ***Affymetrix ID*** | ***Survival outcome*** | ***Basal-like*** | | ***Lumina A*** | | ***Lumina B*** | | ***HER2+*** | |
| --- | --- | --- | --- | --- | --- | --- | --- | --- | --- | --- |
|  |  |  | ***HR (95% CI)*** | ***p-value*** | ***HR (95% CI)*** | ***p-value*** | ***HR (95% CI)*** | ***p-value*** | ***HR (95% CI)*** | ***p-value*** |
| ***TOB1*** | **202704_at** | RFS | **1.50 (1.16 - 1.93)** | **0.0017** | 1.11 (0.94 - 1.32) | 0.2194 | 1.03 (0.85 - 1.25) | 0.7438 | 0.92 (0.62 - 1.34) | 0.6497 |
|  |  | OS | 1.15 (0.71 - 1.89) | 0.5677 | 1.29 (0.90 - 1.83) | 0.1626 | 1.16 (0.80 - 1.69) | 0.4234 | 0.82 (0.43 - 1.56) | 0.5444 |
|  |  | DMFS | 1.37 (0.82 - 2.29) | 0.2228 | 1.43 (1.07 - 1.91) | 0.0156 | 1.02 (0.71 - 1.44) | 0.9335 | 0.65 (0.34 - 1.21) | 0.169 |
|  |  | PPS | 0.88 (0.49 - 1.59) | 0.6785 | 1.33 (0.90 - 1.96) | 0.1513 | 1.06 (0.69 - 1.63) | 0.7935 | 0.75 (0.35 - 1.57) | 0.4389 |
| ***TOB2*** | 221496_s_at | RFS | 1.38 (1.07 - 1.78) | 0.0122 | 1.05 (0.88 - 1.24) | 0.6095 | 1.10 (0.90 - 1.33) | 0.3526 | 1.24 (0.84 - 1.82) | 0.2703 |
|  |  | OS | 1.01 (0.62 - 1.65) | 0.9742 | 0.82 (0.58 - 1.17) | 0.2805 | 0.96 (0.66 - 1.39) | 0.8218 | 1.02 (0.53 - 1.94) | 0.962 |
|  |  | DMFS | 1.14 (0.69 - 1.90) | 0.6057 | 0.90 (0.68 - 1.20) | 0.4762 | 0.98 (0.69 - 1.39) | 0.9052 | 1.24 (0.67 - 2.31) | 0.4947 |
|  |  | PPS | 1.19 (0.66 - 2.14) | 0.5623 | 0.80 (0.54 - 1.18) | 0.2675 | 0.99 (0.64 - 1.52) | 0.9562 | 1.10 (0.52 - 2.32) | 0.7986 |
|  | 222243_s_at | RFS | 0.95 (0.74 - 1.23) | 0.7133 | 0.86 (0.72 - 1.02) | 0.0763 | 0.95 (0.79 - 1.15) | 0.6268 | 1.65 (1.12 - 2.43) | 0.0108 |
|  |  | OS | 1.11 (0.68 - 1.81) | 0.6851 | 0.90 (0.63 - 1.29) | 0.5701 | 0.91 (0.63 - 1.33) | 0.6316 | 1.74 (0.90 - 3.36) | 0.0953 |
|  |  | DMFS | 0.64 (0.38 - 1.07) | 0.0849 | 1.06 (0.79 - 1.42) | 0.6957 | 0.79 (0.56 - 1.13) | 0.1963 | 1.67 (0.89 - 3.13) | 0.1055 |
|  |  | PPS | 0.70 (0.39 - 1.27) | 0.2395 | 1.41 (0.95 - 2.08) | 0.0832 | 1.22 (0.79 - 1.87) | 0.376 | 1.25 (0.59 - 2.69) | 0.5732 |
| ***BTG1*** | 1559975_at | RFS | 0.76 (0.55 - 1.05) | 0.0977 | 0.96 (0.75 - 1.22) | 0.7211 | **0.65 (0.47 - 0.88)** | **0.006** | 0.87 (0.55 - 1.37) | 0.5458 |
|  |  | OS | 1.79 (0.92 - 3.46) | 0.0798 | 1.67 (1.00 - 2.78) | 0.0478 | 0.73 (0.37 - 1.44) | 0.3589 | 0.70 (0.32 - 1.53) | 0.3686 |
|  |  | DMFS | 1.19 (0.59 - 2.41) | 0.6316 | 1.16 (0.67 - 2.00) | 0.5992 | 0.51 (0.26 - 1.03) | 0.0548 | 0.59 (0.27 - 1.26) | 0.1648 |
|  |  | PPS | 2.37 (0.96 - 5.83) | 0.0543 | 1.42 (0.80 - 2.53) | 0.226 | 0.76 (0.36 - 1.60) | 0.4637 | 0.97 (0.42 - 2.26) | 0.9434 |
|  | 200920_s_at | RFS | 1.19 (0.93 - 1.54) | 0.1672 | 1.17 (0.98 - 1.38) | 0.0762 | 1.15 (0.95 - 1.39) | 0.1643 | 1.39 (0.95 - 2.05) | 0.0908 |
|  |  | OS | 0.75 (0.46 - 1.23) | 0.2565 | 0.81 (0.57 - 1.15) | 0.2388 | **0.61 (0.42 - 0.89)** | **0.0092** | 1.00 (0.52 - 1.90) | 0.9904 |
|  |  | DMFS | 0.67 (0.40 - 1.11) | 0.117 | 0.89 (0.67 - 1.19) | 0.447 | **0.59 (0.41 - 0.84)** | **0.0032** | 0.85 (0.46 - 1.59) | 0.613 |
|  |  | PPS | 1.74 (0.96 - 3.16) | 0.0668 | 0.83 (0.56 - 1.23) | 0.3617 | 0.86 (0.56 - 1.33) | 0.5059 | 1.18 (0.56 - 2.50) | 0.6656 |
|  | 200921_s_at | RFS | 0.81 (0.63 - 1.05) | 0.1083 | 1.05 (0.89 - 1.25) | 0.5586 | 0.89 (0.73 - 1.07) | 0.2211 | 1.33 (0.90 - 1.95) | 0.149 |
|  |  | OS | 0.70 (0.43 - 1.15) | 0.1586 | 1.06 (0.74 - 1.50) | 0.7615 | 0.78 (0.54 - 1.12) | 0.1777 | 1.76 (0.90 - 3.42) | 0.0923 |
|  |  | DMFS | 0.64 (0.38 - 1.07) | 0.0827 | 0.87 (0.65 - 1.15) | 0.3287 | **0.56 (0.39 - 0.80)** | **0.0012** | 1.24 (0.67 - 2.32) | 0.4896 |
|  |  | PPS | 0.94 (0.52 - 1.67) | 0.8214 | 1.29 (0.87 - 1.90) | 0.2037 | 0.90 (0.59 - 1.38) | 0.6235 | **3.22 (1.44 - 7.19)** | **0.0029** |
| ***BTG2*** | 201235_s_at | RFS | **0.67 (0.52 - 0.87)** | **0.0021** | **0.65 (0.55 - 0.78)** | **1.20E-06** | 0.80 (0.66 - 0.97) | 0.0238 | **0.44 (0.29 - 0.66)** | **4.10E-05** |
|  |  | OS | 0.84 (0.52 - 1.38) | 0.4998 | 0.66 (0.46 - 0.94) | 0.022 | 1.17 (0.81 - 1.69) | 0.406 | 0.45 (0.23 - 0.88) | 0.0174 |
|  |  | DMFS | 0.59 (0.35 - 0.99) | 0.0425 | **0.66 (0.49 - 0.88)** | **0.0045** | 1.00 (0.71 - 1.43) | 0.9836 | 0.72 (0.38 - 1.34) | 0.2954 |
|  |  | PPS | 1.32 (0.73 - 2.38) | 0.349 | 0.74 (0.50 -1.09) | 0.1294 | 1.15 (0.75 - 1.76) | 0.5309 | 0.96 (0.45 - 2.02) | 0.9079 |
|  | 201236_s_at | RFS | **0.59 (0.46 - 0.77)** | **5.60E-05** | **0.62 (0.52 - 0.73)** | **3.50E-08** | **0.68 (0.56 - 0.82)** | **6.90E-05** | 0.49 (0.33 - 0.74) | 0.00043 |
|  |  | OS | 0.65 (0.40 - 1.07) | 0.0891 | **0.59 (0.41 - 0.85)** | **0.0043** | 0.87 (0.60 - 1.26) | 0.4514 | 0.79 (0.41 - 1.50) | 0.4626 |
|  |  | DMFS | 0.59 (0.35 - 0.99) | 0.0431 | 0.75 (0.56 - 1.00) | 0.0501 | 0.83 (0.58 - 1.18) | 0.3053 | 0.77 (0.41 - 1.43) | 0.4067 |
|  |  | PPS | 1.11 (0.62 - 1.99) | 0.7273 | 0.75 (0.51 - 1.11) | 0.1504 | 1.15 (0.75 - 1.76) | 0.5295 | 0.99 (0.47 - 2.08) | 0.9708 |
| ***BTG3*** | 205548_s_at | RFS | 0.99 (0.77 - 1.29) | 0.948 | 1.05 (0.89 - 1.25) | 0.5694 | 0.88 (0.72 - 1.06) | 0.1788 | 0.72 (0.49 - 1.06) | 0.094 |
|  |  | OS | 0.99 (0.60 - 1.61) | 0.9555 | 1.13 (0.80 - 1.61) | 0.4938 | 1.11 (0.77 - 1.61) | 0.5677 | 0.51 (0.26 - 1.00) | 0.0466 |
|  |  | DMFS | 1.31 (0.79 - 2.18) | 0.2972 | 1.24 (0.93 - 1.66) | 0.1344 | 1.08 (0.76 - 1.53) | 0.6779 | 0.72 (0.39 - 1.34) | 0.3006 |
|  |  | PPS | 0.78 (0.43 - 1.39) | 0.3956 | 1.12 (0.76 - 1.65) | 0.5737 | 0.93 (0.61 - 1.44) | 0.7547 | 1.29 (0.61 - 2.72) | 0.5043 |
|  | 213134_x_at | RFS | 1.11 (0.86 - 1.43) | 0.4235 | 1.10 (0.93 - 1.31) | 0.2638 | 1.24 (1.02 - 1.50) | 0.0289 | 0.75 (0.51 - 1.10) | 0.1432 |
|  |  | OS | 0.70 (0.43 - 1.15) | 0.1608 | 1.10 (0.77 - 1.56) | 0.6144 | 1.47 (1.01 - 2.14) | 0.0415 | 0.59 (0.30 - 1.15) | 0.1156 |
|  |  | DMFS | 1.37 (0.82 - 2.28) | 0.2222 | 1.16 (0.87 - 1.55) | 0.3002 | 1.28 (0.90 - 1.82) | 0.1706 | 0.58 (0.31 - 1.10) | 0.0929 |
|  |  | PPS | 0.97 (0.54 - 1.74) | 0.9305 | 1.14 (0.78 - 1.69) | 0.4963 | 1.16 (0.76 - 1.78) | 0.4989 | 0.68 (0.32 - 1.43) | 0.3032 |
|  | 215425_at | RFS | 0.98 (0.76 - 1.26) | 0.8533 | 0.87 (0.74 - 1.03) | 0.1171 | 1.06 (0.88 - 1.29) | 0.5354 | 1.11 (0.76 - 1.62) | 0.5989 |
|  |  | OS | 0.82 (0.50 - 1.34) | 0.4272 | 0.92 (0.65 - 1.31) | 0.6418 | 1.04 (0.72 - 1.50) | 0.8452 | 1.60 (0.83 - 3.09) | 0.1547 |
|  |  | DMFS | 0.94 (0.57 - 1.56) | 0.8135 | 0.95 (0.72 - 1.27) | 0.7525 | 1.52 (1.06 - 2.17) | 0.0204 | 1.55 (0.82 - 2.91) | 0.173 |
|  |  | PPS | 1.27 (0.71 - 2.28) | 0.4265 | 1.11 (0.75 - 1.64) | 0.6091 | 1.00 (0.65 - 1.54) | 0.998 | 1.69 (0.80 - 3.56) | 0.1667 |
| ***BTG4*** | 220766_at | RFS | 0.83 (0.65 - 1.07) | 0.1571 | 0.83 (0.70 - 0.99) | 0.034 | **0.76 (0.63 - 0.92)** | **0.0056** | 0.86 (0.58 - 1.26) | 0.4289 |
|  |  | OS | 0.75 (0.45 - 1.22) | 0.2443 | 1.15 (0.81 - 1.63) | 0.4452 | 0.70 (0.48 - .03) | 0.0691 | 0.94 (0.50 - 1.80) | 0.8627 |
|  |  | DMFS | 0.64 (0.38 - 1.07) | 0.0859 | 1.12 (0.84 - 1.49) | 0.4298 | 1.36 (0.96 - 1.94) | 0.0859 | 1.40 (0.75 - 2.62) | 0.2921 |
|  |  | PPS | 0.61 (0.34 - 1.11) | 0.1027 | 1.17 ( 0.79 - 1.72) | 0.4322 | 0.86 (0.55 - 1.33) | 0.4911 | 1.08 (0.51 - 2.27) | 0.8411 |

HR, hazard ratio; CI, confidence interval; OS, overall survival; RFS, relapse free survival; DMFS, distant metastasis free survival; PPS, post progression survival. All of the data were obtained from the Kaplan-Meier Plotter database. The data with statistical significance were marked in red.

**Table B. Survival analyses of *APRO* family in lung cancer.**

| ***Gene*** | ***Affymetrix ID*** | ***Survival outcome*** | ***Lung Adenocarcinoma*** | | | ***Squamous Cell Lung Carcinoma*** | | |
| --- | --- | --- | --- | --- | --- | --- | --- | --- |
|  |  |  | ***HR*** | ***95% CI*** | ***p-value*** | ***HR*** | ***95% CI*** | ***p-value*** |
| ***TOB1*** | **202704_at** | **Overall Survival** | **0.58** | **0.46 - 0.73** | **5.0E-06** | 1.08 | 0.85 - 1.37 | 0.5193 |
|  |  | Post Progression Survival | 0.66 | 0.41 - 1.06 | 0.0867 | 0.85 | 0.31 - 2.37 | 0.7572 |
| ***TOB2*** | 221496_s_at | Overall Survival | 1.28 | 1.01 - 1.61 | 0.0389 | 1.1 | 0.87 - 1.40 | 0.4141 |
|  |  | Post Progression Survival | 1.53 | 0.96 - 2.46 | 0.0743 | 0.61 | 0.20 - 1.79 | 0.3612 |
|  | **222243_s_at** | **Overall Survival** | **0.41** | **0.32 - 0.52** | **2.0E-13** | 0.79 | 0.63 - 1.01 | 0.0559 |
|  |  | Post Progression Survival | 0.93 | 0.58 - 1.48 | 0.7496 | 0.54 | 0.19 - 1.54 | 0.2415 |
| ***BTG1*** | **1559975_at** | **Overall Survival** | **0.59** | **0.46 - 0.75** | **2.0E-05** | 1.19 | 0.88 - 1.63 | 0.2595 |
|  |  | Post Progression Survival | 0.91 | 0.56 - 1.48 | 0.6976 | 0.99 | 0.36 - 2.75 | 0.9883 |
|  | **200920_s_at** | **Overall Survival** | **1.64** | **1.29 - 2.08** | **5.0E-05** | 0.8 | 0.63 - 1.01 | 0.0630 |
|  |  | Post Progression Survival | 1.07 | 0.67 - 1.71 | 0.7786 | 0.85 | 0.31 - 2.37 | 0.7572 |
|  | **200921_s_at** | **Overall Survival** | **0.57** | **0.45 - 0.72** | **2.3E-06** | 0.87 | 0.69 - 1.11 | 0.2599 |
|  |  | Post Progression Survival | 0.84 | 0.53 - 1.35 | 0.4798 | 0.85 | 0.31 - 2.37 | 0.7572 |
| ***BTG2*** | **201235_s_at** | **Overall Survival** | **1.46** | **1.15 - 1.85** | **0.0017** | 0.77 | 0.61 - 0.98 | 0.0308 |
|  |  | Post Progression Survival | 1.48 | 0.93 - 2.37 | 0.0953 | 1.86 | 0.60 - 5.72 | 0.2736 |
|  | **201236_s_at** | **Overall Survival** | **0.49** | **0.39 - 0.63** | **4.9E-09** | 0.85 | 0.67 - 1.08 | 0.1756 |
|  |  | Post Progression Survival | 0.66 | 0.42 - 1.06 | 0.0836 | 0.85 | 0.31 - 2.37 | 0.7572 |
| ***BTG3*** | **205548_s_at** | **Overall Survival** | **0.73** | **0.58 - 0.92** | **0.0083** | 1.01 | 0.80 - 1.28 | 0.9221 |
|  |  | Post Progression Survival | 0.73 | 0.45 - 1.16 | 0.1754 | 1.09 | 0.38 - 3.11 | 0.8659 |
|  | **213134_x_at** | **Overall Survival** | **0.73** | **0.58 - 0.92** | **0.0078** | 1.06 | 0.84 - 1.35 | 0.6091 |
|  |  | Post Progression Survival | 0.79 | 0.50 - 1.27 | 0.3295 | 0.35 | 0.11 - 1.06 | 0.0531 |
|  | **215425_at** | **Overall Survival** | **1.43** | **1.13 - 1.82** | **0.0026** | 1.12 | 0.88 - 1.42 | 0.3488 |
|  |  | Post Progression Survival | 1.35 | 0.84 - 2.17 | 0.209 | 1.17 | 0.42 - 3.26 | 0.7628 |
| ***BTG4*** | **220766_at** | **Overall Survival** | **1.52** | **1.20 - 1.92** | **0.00038** | 0.95 | 0.75 - 1.20 | 0.6736 |
|  |  | Post Progression Survival | 1.18 | 0.74 - 1.89 | 0.4864 | 1.04 | 0.36 - 3.02 | 0.9383 |

HR, hazard ratio; CI, confidence interval. All of the data were obtained from the Kaplan-Meier Plotter database. The data with statistical significance were marked in red.

**Table C. Survival analyses of *APRO* family in prostate cancer.**

| ***Gene*** | ***Dataset*** | ***Probe ID*** | ***Survival outcome*** | ***HR*** | ***95% CI*** | ***p-value*** |
| --- | --- | --- | --- | --- | --- | --- |
| ***TOB1*** | GSE16560 | DAP4_5602 | Overall Survival | 1.16 | 0.83 - 1.61 | 0.378088 |
| ***TOB2*** | GSE16560 | DAP1_1269 | Overall Survival | 1.17 | 0.95 - 1.44 | 0.131221 |
| ***BTG1*** | GSE16560 | DAP3_3042 | Overall Survival | 1.35 | 0.97 - 1.87 | 0.0732595 |
| ***BTG2*** | GSE16560 | DAP4_4295 | Overall Survival | 1.11 | 0.89 - 1.39 | 0.333981 |
| ***BTG3*** | GSE16560 | DAP1_0205 | Overall Survival | 1.05 | 0.75 - 1.47 | 0.762764 |

HR, hazard ratio; CI, confidence interval. All of the data were obtain from the PrognScan database.

**Table D. Survival analyses of *APRO* family in colorectal cancer.**

| ***Gene*** | ***Dataset*** | ***Probe ID*** | ***Survival outcome*** | ***HR*** | ***95% CI*** | ***p-value*** |
| --- | --- | --- | --- | --- | --- | --- |
| ***TOB1*** | GSE12945 | 202704_at | Disease Free Survival | 1.59 | 0.46 - 5.50 | 0.467869 |
|  | GSE12945 | 202704_at | Overall Survival | 1.10 | 0.51 - 2.35 | 0.809039 |
|  | GSE17536 | 202704_at | Overall Survival | 1.35 | 0.75 - 2.44 | 0.316417 |
|  | GSE17536 | 228834_at | Disease Free Survival | 0.91 | 0.68 - 1.23 | 0.543419 |
|  | GSE17536 | 202704_at | Disease Free Survival | 1.76 | 0.75 - 4.13 | 0.190167 |
|  | GSE17536 | 202704_at | Disease Specific Survival | 1.36 | 0.69 - 2.68 | 0.372847 |
|  | GSE17536 | 228834_at | Overall Survival | 0.94 | 0.75 - 1.18 | 0.589621 |
|  | GSE17536 | 228834_at | Disease Specific Survival | 0.89 | 0.70 - 1.15 | 0.380554 |
|  | GSE14333 | 202704_at | Disease Free Survival | 1.53 | 0.80 - 2.90 | 0.195483 |
|  | GSE14333 | 228834_at | Disease Free Survival | 0.96 | 0.75 - 1.22 | 0.727342 |
|  | GSE17537 | 202704_at | Disease Free Survival | 1.80 | 0.72 - 4.49 | 0.206259 |
|  | GSE17537 | 228834_at | Disease Free Survival | 0.87 | 0.63 - 1.22 | 0.426776 |
|  | GSE17537 | 202704_at | Disease Specific Survival | 2.16 | 0.73 - 6.41 | 0.165372 |
|  | GSE17537 | 228834_at | Disease Specific Survival | 0.81 | 0.55 - 1.18 | 0.261352 |
|  | GSE17537 | 228834_at | Overall Survival | 0.89 | 0.66 - 1.20 | 0.4293 |
|  | GSE17537 | 202704_at | Overall Survival | 1.54 | 0.64 - 3.69 | 0.337434 |
| ***TOB2*** | GSE12945 | 222243_s_at | Disease Free Survival | 0.92 | 0.09 - 9.42 | 0.947102 |
|  | GSE12945 | 222243_s_at | Overall Survival | 0.84 | 0.19 - 3.67 | 0.818083 |
|  | GSE12945 | 221496_s_at | Disease Free Survival | 0.68 | 0.03 - 14.84 | 0.80635 |
|  | GSE12945 | 221496_s_at | Overall Survival | 1.48 | 0.25 - 8.95 | 0.668293 |
|  | GSE17536 | 234720_s_at | Disease Specific Survival | 0.89 | 0.22 - 3.57 | 0.874792 |
|  | GSE17536 | 222243_s_at | Disease Specific Survival | 1.72 | 0.75 - 3.93 | 0.198433 |
|  | GSE17536 | 225309_at | Overall Survival | 0.85 | 0.40 - 1.83 | 0.686173 |
|  | GSE17536 | 225309_at | Disease Specific Survival | 0.64 | 0.26 - 1.54 | 0.314668 |
|  | GSE17536 | 234720_s_at | Overall Survival | 0.74 | 0.22 - 2.46 | 0.626942 |
|  | GSE17536 | 222243_s_at | Overall Survival | 1.58 | 0.77 - 3.23 | 0.211758 |
|  | GSE17536 | 225309_at | Disease Free Survival | 0.79 | 0.26 - 2.36 | 0.669553 |
|  | GSE17536 | 234720_s_at | Disease Free Survival | 0.77 | 0.14 - 4.12 | 0.756981 |
|  | GSE17536 | 221496_s_at | Disease Specific Survival | 1.25 | 0.63 - 2.48 | 0.523758 |
|  | GSE17536 | 222243_s_at | Disease Free Survival | 1.87 | 0.69 - 5.07 | 0.219611 |
|  | GSE17536 | 221496_s_at | Overall Survival | 1.07 | 0.59 - 1.93 | 0.82438 |
|  | GSE17536 | 221496_s_at | Disease Free Survival | 1.31 | 0.56 - 3.08 | 0.537523 |
|  | GSE14333 | 225309_at | Disease Free Survival | 1.05 | 0.65 - 1.68 | 0.841201 |
|  | GSE14333 | 234720_s_at | Disease Free Survival | 1.04 | 0.83 - 1.31 | 0.714963 |
|  | GSE14333 | 222243_s_at | Disease Free Survival | 1.08 | 0.49 - 2.41 | 0.84248 |
|  | GSE14333 | 221496_s_at | Disease Free Survival | 0.98 | 0.84 - 1.14 | 0.781503 |
|  | GSE17537 | 222243_s_at | Overall Survival | 1.27 | 0.40 - 4.03 | 0.690337 |
|  | GSE17537 | 234720_s_at | Disease Free Survival | 6.18 | 0.36 - 105.65 | 0.208494 |
|  | GSE17537 | 222243_s_at | Disease Free Survival | 1.57 | 0.44 - 5.64 | 0.48627 |
|  | GSE17537 | 221496_s_at | Overall Survival | 0.45 | 0.18 - 1.11 | 0.0828016 |
|  | GSE17537 | 225309_at | Disease Free Survival | 1.10 | 0.30 - 3.98 | 0.882569 |
|  | GSE17537 | 234720_s_at | Disease Specific Survival | 2.26 | 0.07 - 75.15 | 0.647351 |
|  | GSE17537 | 222243_s_at | Disease Specific Survival | 2.69 | 0.54 - 13.46 | 0.228195 |
|  | GSE17537 | 225309_at | Disease Specific Survival | 1.33 | 0.24 - 7.19 | 0.74312 |
|  | GSE17537 | 221496_s_at | Disease Free Survival | 0.37 | 0.12 - 1.09 | 0.0713466 |
|  | GSE17537 | 221496_s_at | Disease Specific Survival | 0.59 | 0.18 - 1.89 | 0.370347 |
|  | GSE17537 | 225309_at | Overall Survival | 0.89 | 0.27 - 2.90 | 0.846658 |
|  | GSE17537 | 234720_s_at | Overall Survival | 1.01 | 0.07 - 15.04 | 0.993691 |
| ***BTG1*** | GSE12945 | 200921_s_at | Disease Free Survival | 0.69 | 0.23 - 2.06 | 0.504722 |
|  | GSE12945 | 200921_s_at | Overall Survival | 1.49 | 0.67 - 3.27 | 0.325806 |
|  | GSE12945 | 200920_s_at | Disease Free Survival | 0.96 | 0.42 - 2.18 | 0.929121 |
|  | GSE12945 | 200920_s_at | Overall Survival | 1.35 | 0.82 - 2.24 | 0.235767 |
|  | GSE17536 | 1559975_at | Disease Free Survival | 1.31 | 0.19 - 8.87 | 0.780927 |
|  | GSE17536 | 200920_s_at | Disease Free Survival | 1.76 | 0.85 - 3.67 | 0.13023 |
|  | GSE17536 | 200921_s_at | Disease Specific Survival | 1.62 | 0.79 - 3.32 | 0.186 |
|  | GSE17536 | 200921_s_at | Overall Survival | 1.40 | 0.75 - 2.59 | 0.291363 |
|  | GSE17536 | 200920_s_at | Disease Specific Survival | 1.29 | 0.71 - 2.36 | 0.406391 |
|  | GSE17536 | 1559975_at | Overall Survival | 0.85 | 0.21 - 3.43 | 0.814663 |
|  | GSE17536 | 200921_s_at | Disease Free Survival | 2.95 | 1.21 - 7.20 | 0.0176854 |
|  | GSE17536 | 1559975_at | Disease Specific Survival | 1.02 | 0.21 - 5.02 | 0.979532 |
|  | GSE17536 | 200920_s_at | Overall Survival | 1.12 | 0.66 - 1.89 | 0.6757 |
|  | GSE14333 | 1559975_at | Disease Free Survival | 1.11 | 0.82 - 1.50 | 0.510737 |
|  | GSE14333 | 200920_s_at | Disease Free Survival | 1.21 | 0.70 - 2.09 | 0.501188 |
|  | GSE14333 | 200921_s_at | Disease Free Survival | 2.31 | 1.02 - 5.26 | 0.0451077 |
|  | GSE17537 | 200920_s_at | Disease Specific Survival | 1.51 | 0.46 - 4.94 | 0.498039 |
|  | GSE17537 | 1559975_at | Disease Free Survival | 0.31 | 0.02 - 3.91 | 0.363637 |
|  | GSE17537 | 1559975_at | Disease Specific Survival | 0.34 | 0.01 - 10.72 | 0.543631 |
|  | GSE17537 | 200921_s_at | Overall Survival | 2.29 | 0.90 - 5.82 | 0.0822224 |
|  | GSE17537 | 1559975_at | Overall Survival | 0.27 | 0.02 - 3.21 | 0.302404 |
|  | GSE17537 | 200921_s_at | Disease Free Survival | 2.90 | 0.97 - 8.68 | 0.0564255 |
|  | GSE17537 | 200920_s_at | Overall Survival | 2.36 | 0.99 - 5.64 | 0.0539392 |
|  | GSE17537 | 200921_s_at | Disease Specific Survival | 2.92 | 0.77 - 11.05 | 0.11541 |
|  | GSE17537 | 200920_s_at | Disease Free Survival | 1.94 | 0.72 - 5.21 | 0.188741 |
| ***BTG2*** | GSE12945 | 201236_s_at | Disease Free Survival | 0.19 | 0.03 - 1.31 | 0.092158 |
|  | GSE12945 | 201236_s_at | Overall Survival | 0.35 | 0.14 - 0.89 | 0.0265214 |
|  | GSE12945 | 201235_s_at | Disease Free Survival | 0.23 | 0.01 - 3.79 | 0.304132 |
|  | GSE12945 | 201235_s_at | Overall Survival | 0.38 | 0.07 - 2.17 | 0.27768 |
|  | GSE17536 | 201235_s_at | Overall Survival | 1.06 | 0.68 - 1.67 | 0.787451 |
|  | GSE17536 | 201236_s_at | Disease Free Survival | 1.13 | 0.62 - 2.09 | 0.686456 |
|  | GSE17536 | 201235_s_at | Disease Free Survival | 0.98 | 0.52 - 1.85 | 0.956062 |
|  | GSE17536 | 201236_s_at | Disease Specific Survival | 1.25 | 0.76 - 2.07 | 0.379213 |
|  | GSE17536 | 201235_s_at | Disease Specific Survival | 1.01 | 0.60 - 1.70 | 0.966502 |
|  | GSE17536 | 201236_s_at | Overall Survival | 1.26 | 0.82 - 1.95 | 0.296837 |
|  | GSE14333 | 201236_s_at | Disease Free Survival | 0.91 | 0.59 - 1.39 | 0.657151 |
|  | GSE14333 | 201235_s_at | Disease Free Survival | 0.89 | 0.62 - 1.29 | 0.536323 |
|  | GSE17537 | 201236_s_at | Disease Specific Survival | 2.01 | 0.77 - 5.27 | 0.153631 |
|  | GSE17537 | 201235_s_at | Disease Free Survival | 0.69 | 0.28 - 1.71 | 0.424119 |
|  | GSE17537 | 201235_s_at | Disease Specific Survival | 0.69 | 0.25 - 1.89 | 0.471265 |
|  | GSE17537 | 201236_s_at | Overall Survival | 2.04 | 0.96 - 4.33 | 0.0654589 |
|  | GSE17537 | 201235_s_at | Overall Survival | 0.96 | 0.43 - 2.14 | 0.919929 |
|  | GSE17537 | 201236_s_at | Disease Free Survival | 2.00 | 0.91 - 4.42 | 0.0865542 |
| ***BTG3*** | GSE12945 | 205548_s_at | Overall Survival | 1.21 | 0.49 - 2.99 | 0.685186 |
|  | GSE12945 | 213134_x_at | Disease Free Survival | 0.57 | 0.15 - 2.21 | 0.419752 |
|  | GSE12945 | 213134_x_at | Overall Survival | 1.15 | 0.49 - 2.70 | 0.755391 |
|  | GSE12945 | 215425_at | Disease Free Survival | 10.72 | 1.30 - 88.68 | 0.0277363 |
|  | GSE12945 | 205548_s_at | Disease Free Survival | 0.61 | 0.14 - 2.64 | 0.512315 |
|  | GSE12945 | 215425_at | Overall Survival | 1.42 | 0.20 - 10.01 | 0.723468 |
|  | GSE17536 | 215425_at | Disease Specific Survival | 0.91 | 0.65 - 1.28 | 0.591294 |
|  | GSE17536 | 205548_s_at | Disease Free Survival | 1.79 | 0.81 - 3.97 | 0.150109 |
|  | GSE17536 | 1556213_a_at | Disease Free Survival | 0.49 | 0.07 - 3.29 | 0.464957 |
|  | GSE17536 | 215425_at | Overall Survival | 0.98 | 0.73 - 1.30 | 0.86547 |
|  | GSE17536 | 213134_x_at | Disease Specific Survival | 1.26 | 0.69 - 2.30 | 0.460584 |
|  | GSE17536 | 215425_at | Disease Free Survival | 1.22 | 0.82 - 1.81 | 0.336145 |
|  | GSE17536 | 205548_s_at | Disease Specific Survival | 1.23 | 0.68 - 2.23 | 0.497843 |
|  | GSE17536 | 213134_x_at | Overall Survival | 1.31 | 0.77 - 2.22 | 0.320214 |
|  | GSE17536 | 1556213_a_at | Overall Survival | 1.03 | 0.33 - 3.18 | 0.962921 |
|  | GSE17536 | 1556213_a_at | Disease Specific Survival | 1.02 | 0.28 - 3.72 | 0.976041 |
|  | GSE17536 | 205548_s_at | Overall Survival | 1.31 | 0.78 - 2.21 | 0.310886 |
|  | GSE17536 | 213134_x_at | Disease Free Survival | 1.86 | 0.82 - 4.22 | 0.139797 |
|  | GSE14333 | 1556213_a_at | Disease Free Survival | 1.05 | 0.79 - 1.41 | 0.720479 |
|  | GSE14333 | 205548_s_at | Disease Free Survival | 1.90 | 0.95 - 3.80 | 0.0696096 |
|  | GSE14333 | 215425_at | Disease Free Survival | 1.16 | 0.82 - 1.64 | 0.392053 |
|  | GSE14333 | 213134_x_at | Disease Free Survival | 1.39 | 0.73 - 2.64 | 0.318733 |
|  | GSE17537 | 205548_s_at | Disease Specific Survival | 1.81 | 0.58 - 5.65 | 0.306561 |
|  | GSE17537 | 215425_at | Overall Survival | 1.19 | 0.65 - 2.18 | 0.575685 |
|  | GSE17537 | 1556213_a_at | Disease Free Survival | 0.61 | 0.07 - 5.30 | 0.650771 |
|  | GSE17537 | 1556213_a_at | Disease Specific Survival | 3.18 | 0.45 - 22.32 | 0.24365 |
|  | GSE17537 | 215425_at | Disease Free Survival | 1.73 | 0.93 - 3.19 | 0.082699 |
|  | GSE17537 | 213134_x_at | Overall Survival | 1.15 | 0.55 - 2.38 | 0.715459 |
|  | GSE17537 | 215425_at | Disease Specific Survival | 1.56 | 0.62 - 3.96 | 0.348403 |
|  | GSE17537 | 1556213_a_at | Overall Survival | 0.77 | 0.11 - 5.42 | 0.794839 |
|  | GSE17537 | 213134_x_at | Disease Free Survival | 1.17 | 0.53 - 2.58 | 0.70371 |
|  | GSE17537 | 205548_s_at | Overall Survival | 1.12 | 0.54 - 2.32 | 0.759745 |
|  | GSE17537 | 213134_x_at | Disease Specific Survival | 1.96 | 0.59 - 6.54 | 0.271461 |
|  | GSE17537 | 205548_s_at | Disease Free Survival | 1.06 | 0.49 - 2.29 | 0.876678 |
| ***BTG4*** | **GSE17537** | **1554362_at** | **Disease Free Survival** | **0.01** | **0.00 - 0.32** | **0.00864609** |
|  | GSE12945 | 220766_at | Disease Free Survival | 25.92 | 0.38 - 1787.83 | 0.131806 |
|  | GSE12945 | 220766_at | Overall Survival | 6.45 | 0.43 - 96.15 | 0.176 |
|  | GSE17536 | 220766_at | Overall Survival | 0.29 | 0.07 - 1.28 | 0.102648 |
|  | GSE17536 | 1554362_at | Overall Survival | 0.48 | 0.11 - 2.16 | 0.336906 |
|  | GSE17536 | 1554362_at | Disease Specific Survival | 0.55 | 0.10 - 3.09 | 0.498695 |
|  | GSE17536 | 220766_at | Disease Free Survival | 0.38 | 0.05 - 3.11 | 0.367331 |
|  | GSE17536 | 1554362_at | Disease Free Survival | 0.88 | 0.11 - 7.08 | 0.901365 |
|  | GSE17536 | 220766_at | Disease Specific Survival | 0.40 | 0.08 - 2.11 | 0.282011 |
|  | GSE14333 | 220766_at | Disease Free Survival | 0.99 | 0.77 - 1.28 | 0.957569 |
|  | GSE14333 | 1554362_at | Disease Free Survival | 1.30 | 0.88 - 1.94 | 0.190007 |
|  | GSE17537 | 220766_at | Disease Free Survival | 0.16 | 0.01 - 3.74 | 0.252407 |
|  | GSE17537 | 220766_at | Disease Specific Survival | 0.38 | 0.01 - 15.05 | 0.603537 |
|  | GSE17537 | 1554362_at | Disease Specific Survival | 0.03 | 0.00 - 1.05 | 0.053119 |
|  | GSE17537 | 220766_at | Overall Survival | 0.25 | 0.01 - 4.93 | 0.360138 |
|  | GSE17537 | 1554362_at | Overall Survival | 0.16 | 0.01 - 1.63 | 0.120036 |

HR, hazard ratio; CI, confidence interval. All of the data were obtain from the PrognScan database. The data with statistical significance were marked in red.

**Table E. Datasets of *APRO* family in kidney, ovarian, brain and CNS cancer.**

| ***Gene*** | ***Dataset*** | ***Normal (Cases)*** | ***Tumor (Cases)*** | ***Fold change*** | ***t-Test*** | ***p-value*** |
| --- | --- | --- | --- | --- | --- | --- |
| **Kidney Cancer** | |  |  |  |  |  |
| ***BTG1*** | Yusenko | Fetal Kidney (2)/Kidney (3) | Clear Cell Renal Cell Carcinoma (26) | 2.143 | 9.556 | 1.53E-10 |
|  | Lenburg | Kidney (9) | Clear Cell Renal Cell Carcinoma (9) | 2.264 | 6.348 | 5.35E-06 |
|  | Gumz | Kidney (10) | Clear Cell Renal Cell Carcinoma (10) | 2.392 | 6.826 | 1.15E-06 |
|  | Jones | Kidney (23) | Clear Cell Renal Cell Carcinoma (23) | 2.81 | 6.925 | 8.07E-09 |
| ***BTG2*** | Higgins | Kidney (3) | Clear Cell Renal Cell Carcinoma (24)  Clear Cell Renal Cell Carcinoma (10)  Clear Cell Renal Cell Carcinoma (23) | -4.981  -2.503  -2.724 | -14.028  -5.213  -20.804 | 2.44E-10 |
|  | Gumz | Kidney (10) |  |  |  | 7.99E-05 |
| ***BTG4*** | Jones | Kidney (23) |  |  |  | 1.50E-23 |
| **Ovarian Cancer** | |  |  |  |  |  |
| ***BTG1*** | Yoshihara | Peritoneum (10) | Ovarian Serous Adenocarcinoma (43) | -3.161 | -8.041 | 7.22E-08 |
|  | Adib | Ovary (4) | Ovarian Serous Adenocarcinoma (6) | -2.665 | -3.422 | 6.00E-03 |
| ***BTG2*** | Yoshihara | Peritoneum (10) | Ovarian Serous Adenocarcinoma (43) | -6.113 | -9.307 | 3.37E-09 |
|  | Adib | Ovary (4) | Ovarian Serous Adenocarcinoma (6) | -5.547 | -6.245 | 1.29E-04 |
|  | Lu | Ovarian Surface Epithelium (5) | Ovarian Serous Adenocarcinoma (20) | -3.289 | -5.57 | 3.64E-05 |
| ***BTG3*** | Adib | Ovary (4) | Ovarian Serous Adenocarcinoma (6) | 2.441 | 3.788 | 3.00E-03 |
|  | Lu | Ovarian Surface Epithelium (5) | Ovarian Serous Adenocarcinoma (20) | 2.278 | 7.16 | 1.72E-07 |
| ***BTG4*** | Yoshihara | Peritoneum (2) | Ovarian Serous Adenocarcinoma (5) | 8.521 | 8.951 | 1.73E-04 |
| **Brain and CNS Cancer** | |  |  |  |  |  |
| ***TOB1*** | Lee | Neural Stem Cell (3) | Glioblastoma (22) | 3.127 | 6.965 | 9.89E-06 |
| ***TOB2*** | Sun | Brain (23) | Diffuse Astrocytoma (7) | 3.581 | 4.051 | 3.11E-04 |
|  |  | Brain (23) | Anaplastic Astrocytoma (19) | 3.804 | 4.939 | 9.00E-06 |
|  |  | Brain (23) | Oligodendroglioma (50) | 3.606 | 5.115 | 7.52E-06 |
| ***BTG1*** | French | Brain (6) | Anaplastic Oligodendroglioma (23) | 4.028 | 16.113 | 1.03E-14 |
|  |  | Brain (6) | Anaplastic Oligoastrocytoma (4) | 4.07 | 13.598 | 5.39E-05 |
|  | Sun | Brain (23) | Oligodendroglioma (50) | 2.632 | 11.916 | 2.83E-18 |
|  |  | Brain (23) | Glioblastoma (81) | 3.232 | 16.06 | 1.17E-23 |
|  |  | Brain (23) | Anaplastic Astrocytoma (19) | 2.44 | 9.305 | 4.42E-11 |
|  |  | Brain (23) | Diffuse Astrocytoma (7) | 2.335 | 5.253 | 3.68E-04 |
|  | Rickman | Temporal Lobe (6) | Astrocytoma (45) | 2.733 | 7.66 | 1.71E-05 |
|  | Bredel 2 | Brain (4) | Anaplastic Oligodendroglioma (3) | 2.927 | 7.031 | 4.93E-04 |
|  |  | Brain (4) | Glioblastoma (27) | 2.547 | 7.491 | 2.58E-05 |
|  | TCGA | Brain (10) | Brain Glioblastoma (542) | 3.457 | 26.246 | 3.57E-11 |
|  |  | Brain (10) | Glioblastoma (5) | 3.453 | 5.289 | 2.00E-03 |
|  | Gutmann | White Matter (3) | Pilocytic Astrocytoma (8) | 2.063 | 4.284 | 5.00E-03 |
|  | Shai | White Matter (7) | Glioblastoma (27) | 3.524 | 6.153 | 2.21E-05 |
|  |  | White Matter (7) | Astrocytoma (5) | 2.114 | 3.913 | 2.00E-03 |
| ***BTG2*** | French | Brain (6) | Anaplastic Oligodendroglioma (23) | 2.264 | 5.976 | 1.47E-06 |
|  | Bredel 2 | Brain (4) | Glioblastoma (27) | 2.022 | 5.33 | 7.56E-06 |
| ***BTG3*** | Shai | White Matter (7) | Glioblastoma (27) | 2.2 | 6.218 | 2.46E-06 |
|  | Pomeroy | Cerebellum (4) | Atypical Teratoid/Rhabdoid Tumor (5) | 3.334 | 4.084 | 5.00E-03 |
|  | French | Brain (6) | Anaplastic Oligoastrocytoma (4) | 3.369 | 4.166 | 4.00E-03 |
|  | Murat | Brain (4) | Glioblastoma (80) | 3.181 | 10.359 | 1.34E-04 |

All of the datasets were obtain from the Oncomine database.

**Table F. Survival analyses of *APRO* family in kidney, ovarian and brain cancer.**

| ***Gene*** | ***Dataset*** | ***Probe ID*** | ***Survival outcome*** | ***HR*** | ***95% CI*** | ***p-value*** |
| --- | --- | --- | --- | --- | --- | --- |
| **Kidney Cancer** | |  |  |  |  |  |
| ***TOB1*** | E-DKFZ-1 | rzpd.de:huber1:Reporter:IMAGE:773342 | Overall Survival | 0.55 | 0.12 - 2.51 | 0.438465 |
| ***BTG1*** | E-DKFZ-1 | rzpd.de:huber1:Reporter:IMAGE:298268 | Overall Survival | 0.93 | 0.31 - 2.77 | 0.897617 |
| **Ovarian Cancer** | |  |  |  |  |  |
| ***TOB1*** | GSE9891 | 202704_at | Overall Survival | 0.90 | 0.67 - 1.20 | 0.466573 |
|  | GSE9891 | 228834_at | Overall Survival | 0.87 | 0.75 - 1.01 | 0.0722326 |
|  | DUKE-OC | 202704_at | Overall Survival | 0.84 | 0.67 - 1.04 | 0.111536 |
|  | GSE8841 | 15225 | Overall Survival | 1.63 | 0.88 - 3.02 | 0.119076 |
|  | GSE8841 | 17268 | Overall Survival | 1.88 | 1.04 - 3.42 | 0.0372644 |
|  | GSE26712 | 202704_at | Overall Survival | 1.19 | 1.01 - 1.40 | 0.0412799 |
|  | GSE26712 | 202704_at | Disease Free Survival | 1.15 | 0.99 - 1.33 | 0.0732752 |
|  | GSE17260 | A_23_P164179 | Progression Free Survival | 0.74 | 0.54 - 1.00 | 0.0473523 |
|  | GSE17260 | A_32_P148085 | Progression Free Survival | 0.71 | 0.50 - 1.02 | 0.0630697 |
|  | GSE17260 | A_23_P164179 | Overall Survival | 0.87 | 0.59 - 1.30 | 0.50938 |
|  | GSE17260 | A_32_P148085 | Overall Survival | 0.79 | 0.49 - 1.26 | 0.324905 |
|  | GSE14764 | 202704_at | Overall Survival | 0.90 | 0.48 - 1.69 | 0.754102 |
| ***TOB2*** | **DUKE-OC** | **222243_s_at** | **Overall Survival** | **0.63** | **0.49 - 0.81** | **0.000285895** |
|  | GSE9891 | 234720_s_at | Overall Survival | 0.88 | 0.42 - 1.84 | 0.728647 |
|  | GSE9891 | 222243_s_at | Overall Survival | 0.86 | 0.61 - 1.23 | 0.420404 |
|  | GSE9891 | 225309_at | Overall Survival | 1.00 | 0.76 - 1.33 | 0.982979 |
|  | GSE9891 | 221496_s_at | Overall Survival | 0.85 | 0.64 - 1.14 | 0.27829 |
|  | DUKE-OC | 221496_s_at | Overall Survival | 0.84 | 0.67 - 1.05 | 0.127884 |
|  | GSE8841 | 16239 | Overall Survival | 2.99 | 0.95 - 9.48 | 0.0622817 |
|  | GSE8841 | 2733 | Overall Survival | 1.49 | 0.74 - 2.99 | 0.259482 |
|  | GSE8841 | 11192 | Overall Survival | 1.10 | 0.48 - 2.49 | 0.828783 |
|  | GSE26712 | 222243_s_at | Disease Free Survival | 1.29 | 0.98 - 1.70 | 0.0746478 |
|  | GSE26712 | 221496_s_at | Disease Free Survival | 1.06 | 0.84 - 1.33 | 0.625179 |
|  | GSE26712 | 222243_s_at | Overall Survival | 1.26 | 0.93 - 1.71 | 0.137207 |
|  | GSE26712 | 221496_s_at | Overall Survival | 1.03 | 0.80 - 1.32 | 0.818621 |
|  | GSE17260 | A_32_P179676 | Overall Survival | 1.27 | 0.71 - 2.25 | 0.421667 |
|  | GSE17260 | A_23_P335813 | Progression Free Survival | 1.46 | 0.90 - 2.38 | 0.127232 |
|  | GSE17260 | A_32_P179676 | Progression Free Survival | 1.25 | 0.79 - 1.97 | 0.338138 |
|  | GSE17260 | A_23_P335813 | Overall Survival | 1.03 | 0.54 - 1.94 | 0.938255 |
|  | GSE14764 | 221496_s_at | Overall Survival | 0.96 | 0.72 - 1.27 | 0.76278 |
|  | GSE14764 | 222243_s_at | Overall Survival | 1.49 | 0.77 - 2.89 | 0.236307 |
| ***BTG1*** | GSE9891 | 1559975_at | Overall Survival | 1.28 | 0.43 - 3.81 | 0.660215 |
|  | GSE9891 | 200921_s_at | Overall Survival | 1.04 | 0.73 - 1.50 | 0.820091 |
|  | GSE9891 | 200920_s_at | Overall Survival | 1.02 | 0.72 - 1.44 | 0.903576 |
|  | DUKE-OC | 200920_s_at | Overall Survival | 0.75 | 0.59 - 0.97 | 0.0295337 |
|  | DUKE-OC | 200921_s_at | Overall Survival | 0.72 | 0.56 - 0.94 | 0.0139811 |
|  | GSE8841 | 154 | Overall Survival | 0.29 | 0.05 - 1.78 | 0.181032 |
|  | GSE26712 | 200921_s_at | Disease Free Survival | 1.11 | 0.93 - 1.33 | 0.246884 |
|  | GSE26712 | 200920_s_at | Disease Free Survival | 1.06 | 0.92 - 1.23 | 0.436352 |
|  | GSE26712 | 200921_s_at | Overall Survival | 1.10 | 0.90 - 1.34 | 0.34527 |
|  | GSE26712 | 200920_s_at | Overall Survival | 1.00 | 0.86 - 1.17 | 0.988626 |
|  | GSE17260 | A_23_P87560 | Progression Free Survival | 0.60 | 0.39 - 0.93 | 0.0217785 |
|  | GSE17260 | A_23_P87560 | Overall Survival | 0.92 | 0.51 - 1.65 | 0.782891 |
|  | GSE14764 | 200920_s_at | Overall Survival | 0.87 | 0.50 - 1.50 | 0.608028 |
|  | GSE14764 | 200921_s_at | Overall Survival | 0.86 | 0.47 - 1.56 | 0.621178 |
| ***BTG2*** | GSE9891 | 201235_s_at | Overall Survival | 0.72 | 0.54 - 0.97 | 0.0320469 |
|  | GSE9891 | 201236_s_at | Overall Survival | 0.88 | 0.71 - 1.09 | 0.234344 |
|  | DUKE-OC | 201235_s_at | Overall Survival | 1.12 | 0.83 - 1.51 | 0.454857 |
|  | DUKE-OC | 201236_s_at | Overall Survival | 0.83 | 0.68 - 1.02 | 0.0703465 |
|  | GSE8841 | 7086 | Overall Survival | 1.00 | 1.00 - 1.00 | 1 |
|  | GSE26712 | 201235_s_at | Overall Survival | 0.93 | 0.68 - 1.26 | 0.631413 |
|  | GSE26712 | 201236_s_at | Disease Free Survival | 1.17 | 0.99 - 1.37 | 0.0591983 |
|  | GSE26712 | 201235_s_at | Disease Free Survival | 0.96 | 0.73 - 1.27 | 0.786528 |
|  | GSE26712 | 201236_s_at | Overall Survival | 1.13 | 0.95 - 1.35 | 0.167092 |
|  | GSE17260 | A_23_P62901 | Progression Free Survival | 0.80 | 0.64 - 1.01 | 0.0595249 |
|  | GSE17260 | A_23_P62901 | Overall Survival | 0.87 | 0.64 - 1.17 | 0.355276 |
|  | GSE14764 | 201235_s_at | Overall Survival | 1.06 | 0.61 - 1.83 | 0.8333 |
|  | GSE14764 | 201236_s_at | Overall Survival | 0.85 | 0.46 - 1.55 | 0.590843 |
| ***BTG3*** | GSE9891 | 1556213_a_at | Overall Survival | 0.73 | 0.54 - 0.99 | 0.0440199 |
|  | GSE9891 | 215425_at | Overall Survival | 0.87 | 0.71 - 1.07 | 0.191796 |
|  | GSE9891 | 213134_x_at | Overall Survival | 0.87 | 0.69 - 1.10 | 0.245325 |
|  | GSE9891 | 205548_s_at | Overall Survival | 0.87 | 0.70 - 1.10 | 0.244051 |
|  | DUKE-OC | 213134_x_at | Overall Survival | 1.10 | 0.91 - 1.34 | 0.322366 |
|  | DUKE-OC | 215425_at | Overall Survival | 1.22 | 0.92 - 1.61 | 0.171066 |
|  | DUKE-OC | 205548_s_at | Overall Survival | 1.12 | 0.93 - 1.35 | 0.247051 |
|  | GSE26712 | 215425_at | Disease Free Survival | 0.83 | 0.51 - 1.37 | 0.470143 |
|  | GSE26712 | 213134_x_at | Disease Free Survival | 0.94 | 0.79 - 1.11 | 0.452502 |
|  | GSE26712 | 215425_at | Overall Survival | 0.90 | 0.53 - 1.53 | 0.685091 |
|  | GSE26712 | 205548_s_at | Disease Free Survival | 0.89 | 0.75 - 1.05 | 0.156009 |
|  | GSE26712 | 213134_x_at | Overall Survival | 0.90 | 0.75 - 1.08 | 0.268727 |
|  | GSE26712 | 205548_s_at | Overall Survival | 0.87 | 0.73 - 1.05 | 0.1453 |
|  | GSE17260 | A_23_P80068 | Progression Free Survival | 1.04 | 0.79 - 1.37 | 0.790344 |
|  | GSE17260 | A_32_P33723 | Progression Free Survival | 1.02 | 0.82 - 1.28 | 0.839756 |
|  | GSE17260 | A_23_P80068 | Overall Survival | 1.05 | 0.73 - 1.51 | 0.780555 |
|  | GSE17260 | A_32_P33723 | Overall Survival | 0.99 | 0.74 - 1.33 | 0.968771 |
|  | GSE14764 | 215425_at | Overall Survival | 0.98 | 0.46 - 2.13 | 0.968799 |
|  | GSE14764 | 205548_s_at | Overall Survival | 0.79 | 0.47 - 1.31 | 0.351982 |
|  | GSE14764 | 213134_x_at | Overall Survival | 0.99 | 0.55 - 1.79 | 0.965497 |
| ***BTG4*** | GSE9891 | 1554362_at | Overall Survival | 0.67 | 0.16 - 2.83 | 0.583659 |
|  | GSE9891 | 220766_at | Overall Survival | 0.54 | 0.21 - 1.35 | 0.186024 |
|  | DUKE-OC | 220766_at | Overall Survival | 63.59 | 1.37 - 2957.87 | 0.0340449 |
|  | GSE26712 | 220766_at | Overall Survival | 1.34 | 0.78 - 2.32 | 0.287354 |
|  | GSE26712 | 220766_at | Disease Free Survival | 1.28 | 0.78 - 2.10 | 0.328569 |
|  | GSE17260 | A_23_P47322 | Progression Free Survival | 0.98 | 0.74 - 1.29 | 0.873427 |
|  | GSE17260 | A_23_P47322 | Overall Survival | 1.02 | 0.73 - 1.42 | 0.894968 |
|  | GSE14764 | 220766_at | Overall Survival | 1.28 | 0.91 - 1.79 | 0.156827 |
| **Brain cancer** | |  |  |  |  |  |
| ***TOB1*** | **GSE7696** | **202704_at** | **Overall Survival** | **1.49** | **1.11 - 2.01** | **0.00761716** |
|  | GSE4271-GPL96 | 202704_at | Overall Survival | 1.87 | 1.05 - 3.32 | 0.0328414 |
|  | GSE4271-GPL97 | 228834_at | Overall Survival | 1.07 | 0.69 - 1.65 | 0.774429 |
|  | GSE7696 | 228834_at | Overall Survival | 1.17 | 0.89 - 1.55 | 0.265524 |
|  | MGH-glioma | 40631_at | Overall Survival | 1.70 | 0.97 - 2.99 | 0.0631387 |
|  | GSE4412-GPL96 | 202704_at | Overall Survival | 0.65 | 0.27 - 1.54 | 0.326941 |
|  | GSE4412-GPL97 | 228834_at | Overall Survival | 0.77 | 0.49 - 1.22 | 0.265797 |
|  | GSE16581 | 202704_at | Overall Survival | 1.03 | 0.17 - 6.35 | 0.975419 |
|  | GSE16581 | 228834_at | Overall Survival | 1.05 | 0.33 - 3.40 | 0.931448 |
| ***TOB2*** | **GSE7696** | **222243_s_at** | **Overall Survival** | **1.92** | **1.26 - 2.94** | **0.00266087** |
|  | **GSE4412-GPL96** | **221496_s_at** | **Overall Survival** | **0.54** | **0.37 - 0.78** | **0.00132859** |
|  | GSE4271-GPL96 | 222243_s_at | Overall Survival | 0.92 | 0.53 - 1.57 | 0.746285 |
|  | GSE4271-GPL96 | 221496_s_at | Overall Survival | 0.80 | 0.60 - 1.05 | 0.109159 |
|  | GSE4271-GPL97 | 225309_at | Overall Survival | 1.13 | 0.54 - 2.38 | 0.740845 |
|  | GSE4271-GPL97 | 234720_s_at | Overall Survival | 0.96 | 0.62 - 1.49 | 0.858997 |
|  | GSE7696 | 221496_s_at | Overall Survival | 1.51 | 0.99 - 2.32 | 0.0572269 |
|  | GSE7696 | 234720_s_at | Overall Survival | 3.55 | 0.76 - 16.59 | 0.107851 |
|  | GSE7696 | 225309_at | Overall Survival | 1.28 | 0.66 - 2.49 | 0.469809 |
|  | MGH-glioma | 39286_at | Overall Survival | 0.52 | 0.31 - 0.88 | 0.0147206 |
|  | GSE4412-GPL96 | 222243_s_at | Overall Survival | 0.46 | 0.23 - 0.89 | 0.0217499 |
|  | GSE4412-GPL97 | 225309_at | Overall Survival | 1.24 | 0.49 - 3.11 | 0.65394 |
|  | GSE4412-GPL97 | 234720_s_at | Overall Survival | 0.51 | 0.27 - 0.94 | 0.0315587 |
|  | GSE16581 | 234720_s_at | Overall Survival | 4.69 | 0.06 - 354.07 | 0.483715 |
|  | GSE16581 | 222243_s_at | Overall Survival | 0.11 | 0.02 - 0.73 | 0.0222006 |
|  | GSE16581 | 225309_at | Overall Survival | 0.04 | 0.00 - 0.82 | 0.0363823 |
|  | GSE16581 | 221496_s_at | Overall Survival | 0.38 | 0.11 - 1.32 | 0.128798 |
| ***BTG1*** | MGH-glioma | 37294_at | Overall Survival | 1.04 | 0.70 - 1.56 | 0.844546 |
|  | GSE4412-GPL96 | 200920_s_at | Overall Survival | 0.71 | 0.36 - 1.42 | 0.329914 |
|  | GSE4412-GPL96 | 200921_s_at | Overall Survival | 0.54 | 0.25 - 1.15 | 0.1102 |
|  | GSE16581 | 200921_s_at | Overall Survival | 1.31 | 0.35 - 4.99 | 0.689731 |
|  | GSE16581 | 200920_s_at | Overall Survival | 1.17 | 0.33 - 4.12 | 0.802287 |
|  | GSE16581 | 1559975_at | Overall Survival | 8.95 | 0.07 - 1084.35 | 0.370398 |
| ***BTG2*** | **GSE4412-GPL96** | **201235_s_at** | **Overall Survival** | **0.61** | **0.46 - 0.82** | **0.000880908** |
|  | **GSE4412-GPL96** | **201236_s_at** | **Overall Survival** | **0.26** | **0.12 - 0.57** | **0.000907401** |
|  | GSE4271-GPL96 | 201236_s_at | Overall Survival | 0.71 | 0.38 - 1.34 | 0.291438 |
|  | GSE4271-GPL96 | 201235_s_at | Overall Survival | 0.74 | 0.54 - 1.03 | 0.0715866 |
|  | GSE7696 | 201236_s_at | Overall Survival | 1.07 | 0.69 - 1.66 | 0.773024 |
|  | GSE7696 | 201235_s_at | Overall Survival | 1.45 | 0.54 - 3.92 | 0.462519 |
|  | MGH-glioma | 36634_at | Overall Survival | 0.74 | 0.45 - 1.21 | 0.225777 |
|  | GSE16581 | 201236_s_at | Overall Survival | 0.75 | 0.21 - 2.71 | 0.655911 |
|  | GSE16581 | 201235_s_at | Overall Survival | 1.00 | 0.37 - 2.73 | 0.994611 |
| ***BTG3*** | **GSE4271-GPL96** | **213134_x_at** | **Overall Survival** | **3.86** | **1.89 - 7.88** | **0.000206133** |
|  | **GSE4271-GPL96** | **215425_at** | **Overall Survival** | **2.09** | **1.26 - 3.46** | **0.0041454** |
|  | **GSE4271-GPL96** | **205548_s_at** | **Overall Survival** | **3.41** | **1.80 - 6.45** | **0.000167087** |
|  | GSE7696 | 213134_x_at | Overall Survival | 1.29 | 0.85 - 1.96 | 0.24026 |
|  | GSE7696 | 1556213_a_at | Overall Survival | 2.14 | 0.48 - 9.50 | 0.317676 |
|  | GSE7696 | 205548_s_at | Overall Survival | 1.32 | 0.89 - 1.98 | 0.171028 |
|  | GSE7696 | 215425_at | Overall Survival | 1.54 | 0.51 - 4.70 | 0.446273 |
|  | MGH-glioma | 37218_at | Overall Survival | 1.75 | 1.09 - 2.81 | 0.0196949 |
|  | MGH-glioma | 32568_at | Overall Survival | 0.32 | 0.06 - 1.88 | 0.208469 |
|  | GSE4412-GPL96 | 215425_at | Overall Survival | 1.81 | 0.87 - 3.75 | 0.111133 |
|  | GSE4412-GPL96 | 205548_s_at | Overall Survival | 1.29 | 0.59 - 2.84 | 0.523572 |
|  | GSE4412-GPL96 | 213134_x_at | Overall Survival | 1.40 | 0.65 - 3.00 | 0.388046 |
|  | GSE16581 | 215425_at | Overall Survival | 1.56 | 0.22 - 11.03 | 0.654031 |
|  | GSE16581 | 213134_x_at | Overall Survival | 1.93 | 0.21 - 17.83 | 0.563806 |
|  | GSE16581 | 205548_s_at | Overall Survival | 1.37 | 0.16 - 11.61 | 0.77156 |
|  | GSE16581 | 1556213_a_at | Overall Survival | 0.21 | 0.01 - 7.72 | 0.392118 |
| ***BTG4*** | GSE4271-GPL96 | 220766_at | Overall Survival | 1.30 | 0.89 - 1.92 | 0.177616 |
|  | GSE7696 | 220766_at | Overall Survival | 2.45 | 0.36 - 16.67 | 0.359793 |
|  | GSE7696 | 1554362_at | Overall Survival | 0.11 | 0.01 - 1.48 | 0.0969183 |
|  | GSE4412-GPL96 | 220766_at | Overall Survival | 1.11 | 0.72 - 1.72 | 0.629137 |
|  | GSE16581 | 220766_at | Overall Survival | 11.10 | 0.11 - 1075.88 | 0.302226 |
|  | GSE16581 | 1554362_at | Overall Survival | 0.07 | 0.00 - 1642.41 | 0.60949 |

HR, hazard ratio; CI, confidence interval. All of the data were obtain from the PrognScan database. The data with statistical significance were marked in red.
