# Supplementary figures and images for: Expression and prognosis analyses of the Tob/BTG antiproliferative (APRO) protein family in human cancers
Source: PLoS One. 2017 Sep 18;12(9):e0184902. doi: 10.1371/journal.pone.0184902 (PMC5602628; doi:10.1371/journal.pone.0184902)

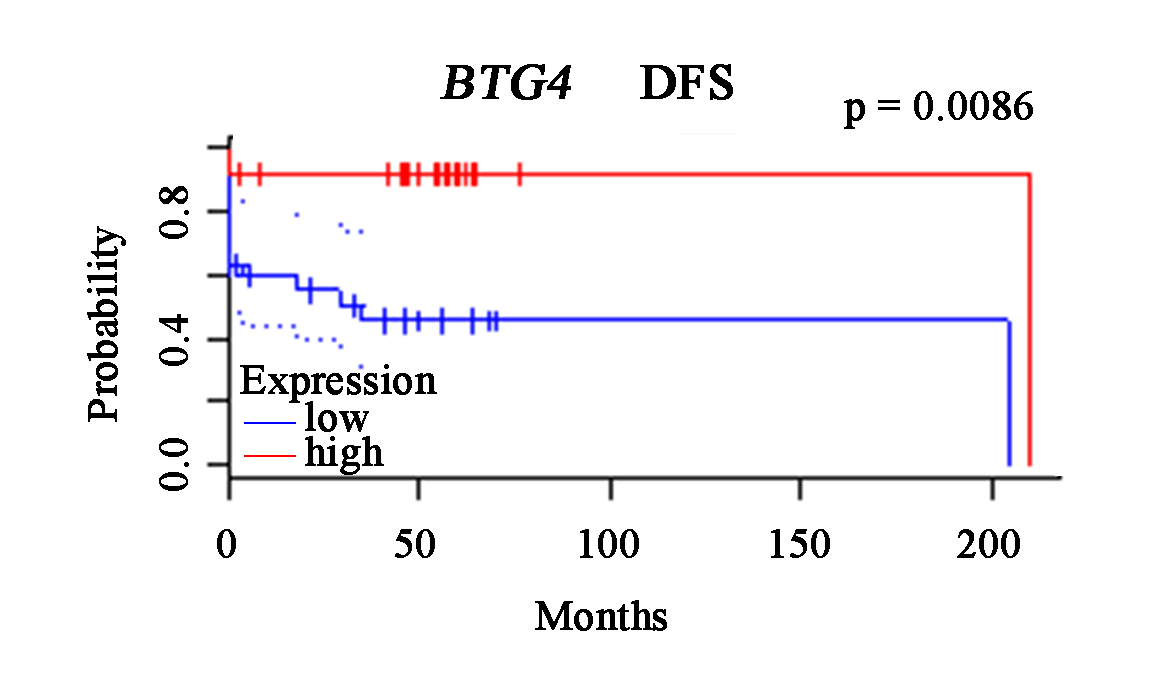

Supplement: S1 Fig — DFS, disease free survival. Survival analysis of BTG4 was obtained from the PrognScan database. (TIF) [file pone.0184902.s002.tif]

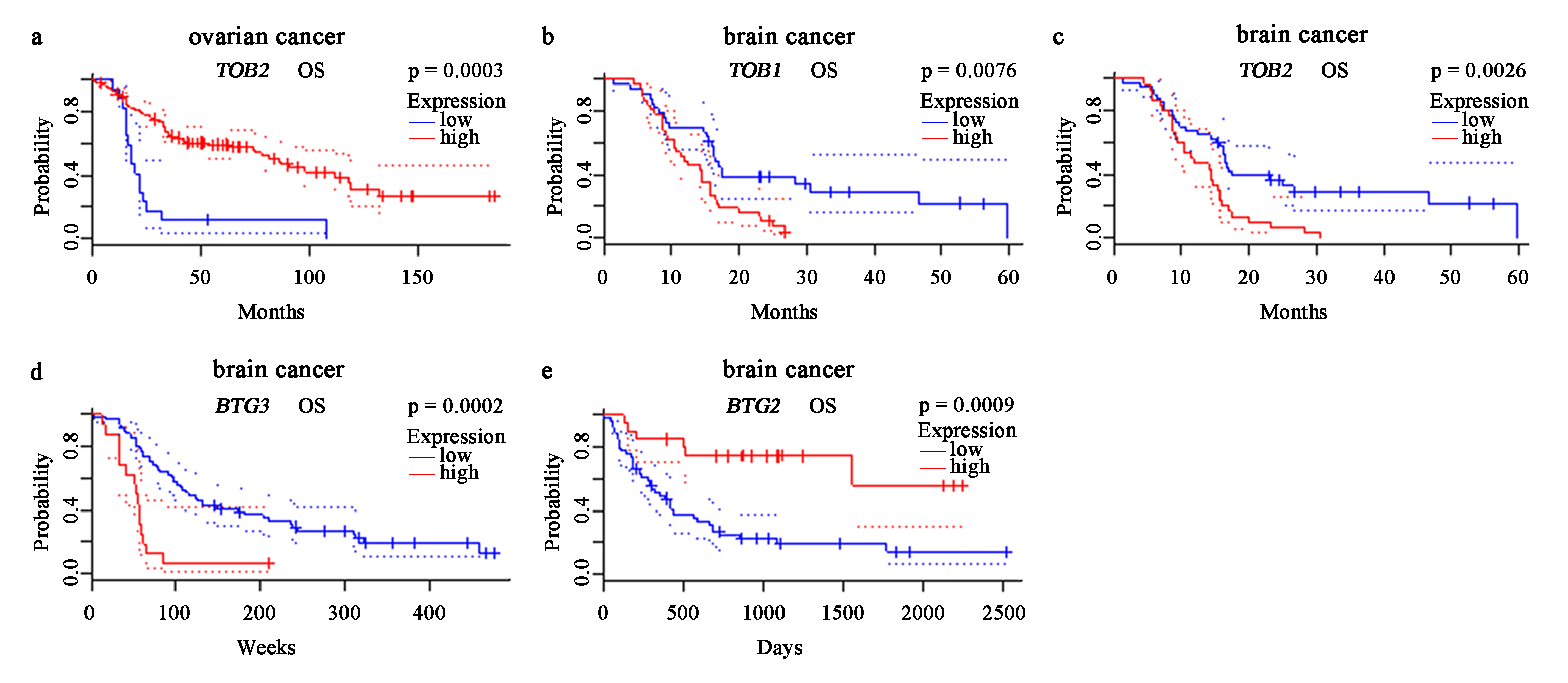

Supplement: S2 Fig — OS, overall survival. (a): survival analysis of TOB2 in ovarian cancer. (b-e): survival analyses of TOB1-2 and BTG2-3 in brain cancer. (TIF) [file pone.0184902.s003.tif]
